# Peers and Homophobic Attitudes in Adolescence: Examining Selection and Influence Processes in Friendships and Antipathies

**DOI:** 10.1007/s10964-020-01298-8

**Published:** 2020-08-13

**Authors:** Chaïm la Roi, Jan Kornelis Dijkstra, Tina Kretschmer, Rūta Savickaitė, René Veenstra

**Affiliations:** 1 Swedish Institute for Social Research (SOFI), Stockholm University, Universitetsvägen 10F, 106 91 Stockholm, Sweden; 2 Institute for Futures Studies, Holländargatan 13, 111 36 Stockholm, Sweden; 3 Department of Sociology, Interuniversity Center for Social Science Theory and Methodology (ICS), University of Groningen, Grote Rozenstraat 31, 9712 TG Groningen, The Netherlands; 4 Department of Pedagogy and Educational Science, University of Groningen, Grote Rozenstraat 38, 9712 TJ Groningen, The Netherlands

**Keywords:** Homophobic attitudes, Peer influence, Negative influence, Attitude dynamics, Stochastic actor-oriented models

## Abstract

Homophobic attitudes and behavior are a widespread problem among adolescents, but what the role of peer relationships such as friendships and antipathies is in shaping these attitudes remains unclear. Therefore, this study examined to what extent homophobic attitudes are influenced by friends’ and foes’ homophobic attitudes, and whether homophobic attitudes serve as a selection criterion for the formation of friendships and antipathies. Participants came from three Dutch high schools across two waves (wave 1 November 2014, wave 2 March/April 2015, ages 11–20, *N* = 1935, 51.5% girls). Stochastic actor-oriented models were estimated for testing hypotheses. The results showed that adolescents adjusted their homophobic attitudes to their friends’ homophobic attitudes, but homophobic attitudes were not consistently related to friendship selection. Further, findings indicated that being dissimilar in homophobic attitudes increased the likelihood to dislike cross-sex peers. Together, the findings suggest that adolescents’ homophobic attitudes were to some extent subject to peer influence, but homophobic attitudes did not steer who adolescents befriended or disliked.

## Introduction

Homophobic attitudes and behavior are a widespread problem among adolescents. Recent studies report that approximately half of all adolescents fall victim to homophobic name-calling over the course of adolescence, with same-sex attracted boys running a particularly high risk (Collier et al. 2013). Being the target of homophobic behavior has detrimental effects on the mental health of lesbian, gay, and bisexual (LGB) (Aragon et al. 2014) as well as heterosexual adolescents (Slaatten et al. 2015). Because homophobic attitudes serve as an important prerequisite for the expression of homophobic behavior in adolescence (Poteat et al. 2013, 2015), a thorough understanding of them is warranted.

Previous research suggests that peers affect the way adolescents think about homosexuality. Adolescents’ homophobic attitudes are similar to those of their friends (Poteat 2007). Whether this similarity is the result of peer influence is unclear. First, peer influence on homophobic attitudes may be confounded by selection processes driven by homophily principles as well as by endogenous network processes that might lead to friends having similar homophobic attitudes. Second, earlier work on this topic conceptualized peer influence as friends growing closer in homophobic attitudes over time (Poteat 2007). However, from studies in the field of attitude dynamics (e.g., Flache and Macy 2011) it can be derived that adolescents that do not like each other may distance themselves from each other in terms of homophobic attitudes, resulting in negative influence. The aim of this study was to fill these two gaps, thereby advancing knowledge on the relevance of peers for adolescents’ homophobic attitudes by empirically distinguishing all these processes.

### Homophobic Attitudes in Adolescence

Adolescence is the period during in which sexuality and sexuality-related matters become salient. Engagement in sexual and romantic behavior is a normative aspect of adolescent development (Collins et al. 2009). Furthermore, most adolescents in Western countries become sexually active during this period (Mercer et al. 2013).

Sexual development in adolescence includes not only actual sexual behaviors, but also sexual identity development (Russell 2005). A problematic component of sexual identity development is the establishment of homophobic attitudes. The salience of sexual orientation has been associated with higher levels of homophobic prejudice among heterosexual adolescents (Poteat et al. 2013). This suggests that adolescents whose sexual orientation is highly important to them are more likely to view others through this categorical lens and develop stereotypes and prejudice related to sexual orientation (Bigler and Liben 2006). Consequently, they may put greater effort into ensuring that their own sexual orientation is known to others. In line with this, using homophobic epithets among adolescents in general and adolescent boys in particular has been identified as a strategy to develop and demonstrate one’s heterosexual, masculine identity to peers (Plummer 2001).

In addition to demonstrating heterosexuality to peers, adolescents may display homophobia as a bullying strategy to acquire social status (Poteat and DiGiovanni 2010). Homophobic bullying is argued to serve two purposes. First, it may lead to the emasculation of its targets, thereby undermining their social status, especially among boys. Second, given the still marginalized and stigmatized position of sexual minority individuals in society, homophobic name-calling may carry greater weight than other means for harassing peers. Although the link between homophobic attitudes and homophobic bullying is strongest in boys, highly prejudiced girls have also been found to use homophobic language as a bullying strategy (Poteat and DiGiovanni 2010). In sum, homophobic attitudes may be salient in adolescents’ lives.

This study focuses on prejudice toward gay males, as this is the group within the population of sexual minority individuals that faces the harshest discrimination. For instance, research among college students found that attitudes are more negative toward gay men than toward lesbian women (Hinrichs and Rosenberg 2002; Swank and Raiz 2010), and that gay men are more often discriminated against than lesbian women (Katz-Wise and Hyde 2012). Furthermore, in the adolescent context, homophobic attitudes were predominantly associated with the victimization of gay male targets (Prati 2012).

### Peers and Homophobic Attitudes

The social salience of homophobic attitudes in adolescence suggests that peers may play a role in the development and expression of these attitudes. In general, peers become increasingly important in adolescence (Bukowski et al. 2018). Because of this and more time spent with peers, adolescents may modify their behavior and attitudes in order to blend in with their peer group. Several mechanisms might account for this. Social learning theory, for instance, postulates that people learn through observing others’ behavior, attitudes, and outcomes of those behaviors (Bandura and Walters 1977). Consequently, adolescents might simply copy the behavior of their peers. Moreover, the theory identifies a number of factors that increase the likelihood for observational learning to occur. For instance, adolescents are more likely to adopt behavior or attitudes of others they feel similar to, or identify with others who possess desired qualities. This latter may be a reason why popular adolescents have been shown to be able to steer classroom norms (Laninga-Wijnen et al. 2018). Moreover, such norms might be steered by reinforcing desired and punishing norm-defying attitudes and behavior. Importantly, adolescents might not only update their opinions because of the social consequences of their own actions, but also by observing how others fare when following or defying the norm (vicarious reinforcement). Within the adolescent peer context, reward and punishment might come in the form of social acceptance and rejection. That is, it is argued in the peer norm literature that socially rewarding normative behavior or attitudes and rejecting behavior or attitudes that are in contrast with the norm, are ways in which such norms are maintained or enforced (Dijkstra et al. 2008).

A complementary argument is provided by subjective group dynamics theory (Abrams et al. 2003), which states that when youth reach adolescence, they become more strongly attuned to norms in the peer group, and more positively evaluate peers expressing opinions that confirm rather than disobey the peer norm. Moreover, the social reasoning developmental perspective adds to this that attunement to peer norms may lead youth to become prejudiced, in particular when fair and just reasoning would be in conflict with peer norms (Rutland et al. 2010). In line with this, experimental studies showed that youth expect that challenging group norms will result in social exclusion (Mulvey et al. 2016). Together, these processes imply that the potentially detrimental social consequences of defying the norm might lead adolescents to adapt their attitudes to those of their peers, leading to a merger of homophobic attitudes in adolescent peer groups. Moreover, an important implication of these literatures is that youth may condone or internalize homophobic attitudes if they perceive this to be an important norm within their peer group, even when they infer this from strategically applied homophobic behavior of their peers. In line, previous research proposes that peers are of substantial significance for the development of attitudes of youth (e.g., Santos et al. 2017; van Zalk et al. 2013).

What is more, research suggests that adolescents’ homophobic attitudes are subject to peer influence. For instance, classroom levels of homophobic attitudes have been found to predict individual level aggression toward male schoolmates perceived to be gay (Prati 2012). Relatedly, a study on Norwegian adolescents revealed that having heard a peer using homophobic name-calling predicted participants’ likelihood to resort to homophobic name-calling themselves (Slaatten et al. 2015). Within the larger peer group, friends have been found to be of particular importance (Lomi et al. 2011). Moreover, previous research points to friends influencing the homophobic attitudes of adolescents. Using multilevel modeling, several researchers have shown that mean levels of homophobic attitudes and behavior within one’s friendship group account for a substantial part of adolescents’ homophobic attitudes (Poteat 2007) and behavior (Birkett and Espelage 2015). Furthermore, homophobic attitudes of friends predicted adolescents’ homophobic attitudes over time, suggesting peer influence (Poteat 2007).

Peer influence regarding homophobic attitudes could also result in other consequences than the merger of attitudes among friends. The expression of homophobic behavior and name-calling toward peers can be used in an effort to maintain and position oneself within adolescent peer groups (McCann et al. 2009; Plummer 2001). A logical extension of this process may be to distance oneself from peers who are accepting of homosexuality in order to pronounce existing differences and stress the portrayal of a heterosexual self-image. And, vice versa, is it feasible that adolescents who are accepting of homosexuality find it important to distinguish themselves from homophobic peers.

These mechanisms are in line with studies on polarization. Social influence has traditionally been defined as a process of opinion averaging (Friedkin and Johnsen 1990), which may indeed comprise an important component of social influence. At the same time, theoretical and simulation studies have concluded that defining social influence as opinion averaging inevitably leads to converging opinions in groups or even societies (Mäs and Flache 2013). Consequently, using such a narrow definition of social influence is not feasible for explaining the persistence of diversity in, or even polarization of opinions (Dandekar et al. 2013; Mäs and Flache 2013). Therefore, social influence might also consist of actively distancing in attitudes, so-called negative influence (Flache and Macy 2011). This negative influence might occur between people who do not like each other (Takács et al. 2016). This idea resonates with balance theory (Heider 1946) and the theory of cognitive dissonance (Festinger 1957), which assert that people prefer to be in a situation of cognitive consistency. Regarding peers that do not like each other (“foes”), this is achieved by disagreeing with people you dislike on issues that are socially salient (Heider 1946). Given the ubiquity of homophobic attitudes in the adolescent peer context (Slaatten et al. 2015), it might be that this is a trait that triggers such a process.

Although negative influence is a straightforward and intuitively appealing micro-process for explaining diversity or polarization of opinions, there is only limited empirical evidence for the existence of it. Most lab experiments testing negative influence returned null findings (Flache et al. 2017; Takács et al. 2016). However, a recent field experiment using Twitter did reveal evidence in line with negative influence (Bail et al. 2018). In that study, a sample of US Twitter users self-identifying as Republican or Democrat were followed over a one-month period. Participants in the treatment condition followed a Twitter bot that retweeted messages from a sample of liberal (for Republican participants) or conservative (for Democratic participants) Twitter accounts. In particular Republican participants that were confronted with liberal messages became substantially more conservative by the end of the study period.

An implication of this relative lack of empirical evidence for negative influence might be that negatively valued peer relationships alone may not be enough to spark negative influence within the adolescent peer context. Youth might simply avoid interacting with peers they dislike, leading to ignorance rather than negative influence. Therefore, it may be necessary to take into account additional conditions that are thought to increase the likelihood for negative influence to occur. Two conditions that may be relevant here are differing in demographic traits and strong opinion dissimilarity (Flache et al. 2017). Using faultline theory (Thatcher and Patel 2011), it could be contended that negative influence occurs only when foes also differ on relevant demographic traits, leading to more strictly delineated and thereby more socially salient groups. The more differences in individual characteristics will be aligned, the more differences between groups of people will be highlighted, meaning that groups are more internally homogenous and contrasts between groups are starker. In such a situation, group differences become more salient, impeding cooperation and increasing tensions between members of different groups. Trait dissimilarity is often used as an important condition for negative influence in theoretical opinion dynamics models, by inferring from balance theory (Heider 1946) and the theory of cognitive dissonance (Festinger 1957) that individuals strive to accentuate disagreement with others if these are too dissimilar (Flache et al. 2017).

A fruitful application of aforementioned conditions for studying negative influence on homophobic attitudes within the adolescent peer context might be to study peers that do not like each other and are of a different sex. To begin, sex is perhaps the most important characteristic for steering peer interactions in adolescence (Bukowski et al. 2018) and delineating groups more generally (Rico et al. 2007). Furthermore, boys on average are more homophobic than girls. By studying cross-sex foes, focus is thus on peers that dislike each other, differ on a relevant demographic trait, and likely differ strongly in the extent to which they are homophobic.

### Selection Mechanisms in Adolescents’ Homophobic Attitudes

Peer influence on homophobic attitudes can only be reliably studied when selection processes are considered. That is, when examining social influence, one should control for selection processes that might serve as an alternative explanation for (dis)similarity in homophobic attitudes between peers. Selection of friends is often based on similarity in certain traits. Similarity in traits might stimulate friendship creation as it leads to increased trust and shared knowledge, eases communication, and fosters mutual understanding, explaining such homophily (McPherson et al. 2001). Selection most likely occurs on attitudes that are socially salient. Homophobic attitudes may occupy such a position in adolescence, given the pervasiveness of homophobic prejudice within the adolescent peer context (Horn 2006) and the strong link between homophobic attitudes and sexual identity development (Poteat et al. 2013). Although homophobic attitudes themselves are a nonvisible trait, their consequences can be visible, for instance through the verbalization of attitudes during conversations, including the use of homophobic epithets, or the display of homophobic behavior. This attests the social salience of homophobic attitudes and enables selection on behavior, another important driver of homophily (McPherson et al. 2001).

When adolescents endorse very different homophobic attitudes, a reversed pattern could occur. That is, in addition to fostering interaction between adolescents with similar homophobic attitudes, dissimilarity in homophobic attitudes may cause adolescents to avoid social interaction or to dislike each other. This is often referred to as the *repulsion hypothesis*, which states that dissimilarity in attitudes leads individuals to evaluate each other negatively (Takács et al. 2016). As with negative influence, the argument for this process was inspired by balance and cognitive dissonance theory (Festinger 1957; Heider 1946), this time using attitude incongruence as a theoretical starting point. In such a situation, cognitive dissonance might be resolved by disliking one another. From a relational perspective, such a process entails the establishment of an antipathy relationship between two individuals with very different homophobic attitudes.

## The Current Study

The aim of this study was to investigate the role of positive (friendships) and negative (foes) peer relationships in the development of homophobic attitudes in adolescence. Regarding influence processes within friendships, because of the pervasiveness and social salience of homophobic attitudes during adolescence, and in line with earlier research, it was expected that over time, adolescents’ homophobic attitudes would become more similar to their friends’ homophobic attitudes (Hypothesis 1). Furthermore, whether negative influence processes with regard to homophobic attitudes could be found between adolescents that dislike one another was examined, testing the following hypothesis: Over time, adolescents’ homophobic attitudes would become more dissimilar to their foes’ homophobic attitudes (Hypothesis 2).

With regard to selection, it was first expected that selection on homophobic attitudes would play a role in the establishment of friendships, leading to the following hypothesis: Adolescents would be more likely to select peers as friends when they are similar in homophobic attitudes (Hypothesis 3). Following the repulsion hypothesis, dissimilarity in homophobic attitudes would lead to disliking between adolescents. Thus, it was expected that adolescents would be more likely to dislike each other when they are dissimilar in homophobic attitudes (Hypothesis 4).

Finally, acknowledging that disliking alone might not be a sufficient condition for negative influence between peers, hypotheses two and four were retested for cross-sex dyads of adolescents that did not like each other by preserving from the antipathy networks only ties between actors of a different sex. This way, it was tested whether negative influence was observed between adolescents that both disliked each other and were of different sex (Hypothesis 2b). An additional benefit of this follow-up analysis is that it enabled us to find out whether or not a “faultline” effect can be detected in network selection between cross-sex peers, in the sense that differing strongly from cross-sex peers in terms of homophobic attitudes increased the likelihood of disliking (Hypothesis 4b).

## Method

### Sample

Data came from *Peers and the Emergence of Adolescent Romance (PEAR)-study*, a Dutch short-term longitudinal study on adolescent romantic and sexual development. The sample used in the present analyses consisted of all students of three high schools located in two municipalities in the northern part of the Netherlands.^1^ The average disposable income in the two municipalities where the schools are located is close to the country average. Both municipalities were homogenous in terms of ethnic composition, with only 3.6% of the population having a non-Western background.

Table 1 provides a sample overview per school. Participants (*N* = 1935, 51.5% girls) were 11 to 20 years of age. Two waves of data were collected. The first wave was collected in November 2014, the second in March and April 2015. Schools sent information about the study and permission forms to parents. Parents who did not want their child to participate in the assessment were asked to return the form. Students were informed at school about the research and gave written consent. Questionnaires were filled in by paper and pencil, within one school hour. Three of the authors were responsible for the data collection. The data were anonymized before the analyses, and questionnaires were completed on a voluntary basis.

**Table 1:** Means and percentages of sociodemographic traits by school

	School 1 (*n* = 756)	School 2 (*n* = 1011)	School 3 (*n* = 167)
Age in years at wave 1 (11–20)	14.76 (1.32)	15.16 (1.76)	14.27(1.37)
Gender			
Boys	55.7%	42.5%	52.1%
Girls	44.3%	57.5%	47.9%
Sexual orientation			
Heterosexual	94.4%	93.5%	94.0%
Sexual minority	5.6%	6.5%	6.0%
Country of birth			
The Netherlands	94.9%	97.3%	96.7%
Turkey	0.6%	0.3%	0.7%
Morocco	0.1%	0.0%	0.0%
Surinam	0.3%	0.2%	0.0%
Dutch Antilles	1.4%	0.2%	0.7%
Indonesia	0.0%	0.0%	0.0%
Other Western	0.7%	0.6%	0.7%
Other non-Western	2.0%	1.4%	1.3%
Country of birth father			
The Netherlands	80.3%	91.1%	95.4%
Turkey	4.8%	1.9%	2.0%
Morocco	0.6%	0.2%	0.0%
Surinam	2.1%	1.2%	0.7%
Dutch Antilles	2.6%	0.2%	1.3%
Indonesia	1.1%	0.7%	0.0%
Other Western	2.7%	2.6%	0.7%
Other non-Western	5.8%	2.1%	0.0%
Country of birth mother			
The Netherlands	84.1%	92.4%	95.4%
Turkey	4.5%	1.5%	0.7%
Morocco	0.9%	0.2%	0.7%
Surinam	1.1%	0.8%	0.0%
Dutch Antilles	2.0%	0.3%	1.3%
Indonesia	0.7%	0.3%	0.7%
Other Western	2.3%	2.7%	0.7%
Other non-Western	4.5%	1.7%	0.7%
SES: type of house			
Detached	39.5%	55.3%	58.0%
Semi-detached	19.1%	26.0%	23.3%
Terraced	39.3%	17.4%	17.3%
Apartment	2.1%	1.3%	1.4%
SES: family holiday frequency			
Seldom to never	11.4%	5.7%	11.3%
Not every year	27.3%	17.5%	19.3%
Once or several times per year	61.3%	76.8%	69.4%

Valid % used. Missing fraction on categorical variables ranged between 0% (gender in Schools 1 and 3) and 8.4% (sexual orientation School 3)

### Measures

#### Homophobic attitudes

These were measured using a scale consisting of eight Likert-type statements, which was developed for the purpose of this study. The most straightforward items from existing scales on homophobic attitudes were selected (Herek 1988; Kuyper 2015; van de Meerendonk et al. 2003; van Wijk et al. 2005), thereby safeguarding comprehensibility by even the youngest participants. The scales showed satisfactory levels of internal consistency (*α* = 0.92). Furthermore, principal component analysis indicated that all items loaded strongly on one factor, with loadings ranging between 0.78 and 0.85. Scale items referred to attitudes toward homosexual men or homosexuality in general. Items included *“*I think it is disgusting when two men kiss”; “I’m getting tired of all the attention for homosexuals*”*. Answering options ranged between *completely disagree* (1) and *completely agree* (5). Higher values on the scale indicated more homophobic attitudes. Considering the low age of some of the participants, statements were formulated straightforwardly. Appendix A provides an overview of the scale items. Appendix B reports findings from an additional sample that (1) demonstrated that the items used in the scale were comprehensible for young participants, (2) confirmed the psychometric properties of the scale, and (3) ascertained its validity.

Participants’ development of homophobic attitudes was examined using the Simulation Investigation for Empirical Network Analysis software package in R (RSiena), version 1.2–12. For this purpose, the continuous homophobic attitudes scale was split into eight categories, each one containing answers on a range of 0.5 points on the original scale.

#### Friendship and antipathy networks

Networks were constructed based on peer nominations for the questions *“*Who are your best friends?*”* and *“*Whom do you not like?*”*. For both questions, participants could nominate up to 24 peers from the same school, resulting in school level networks.

### Control Variables

#### Sex

Participants could rate their sex as girl (0), boy (1), and were provided a fill-in option in case this dichotomy did not apply (“Other, namely ….”), yet this option was not used.

#### Ethnicity

This variable was operationalized as a dichotomy, distinguishing between ethnic majority members (0) and ethnic minority members (1). Participants categorized as ethnic minority background members when either they or at least one of their parents was born in a non-Western country, in line with the definition used in Dutch population registers.^2^

#### Sexual orientation

This was measured using a sexual self-identification question: “What do you think you are?” Answering options were *heterosexual, homosexual*^3^*, bisexual, don’t know*, and *no answer*. *Heterosexual* was coded as zero, *homosexual* or *bisexual* as 1, and *don’t know* or *no answer* as missing.

#### Grade

Information was provided by the school. Its effect was included to control for friendship and antipathy selection due to physical proximity.

#### Class

Information was provided by the school. Its effect was included to control for friendship and antipathy selection due to physical proximity.

### Analytic Strategy

Participants’ development of homophobic attitudes was examined using stochastic actor-oriented models in RSiena, a method that has been developed for the analysis of the longitudinal development of networks and behavior or attitudes (Ripley et al. 2019). The method models change in network constellations or attitudes between two waves of data collection as a sequence of many small changes called “micro-steps”, as in an agent-based model. At each step, one actor in the model can create, maintain, or dissolve one outgoing network tie, or consolidate or change its attitude. These changes can be modeled based on individual characteristics, structural network characteristics, behavioral tendencies, and friends’ and foes’ characteristics (Snijders 2001; Snijders et al. 2010).

Of special interest to this study is the method’s ability to model the co-evolution of multiple networks and attitudes simultaneously (Snijders et al. 2013; Steglich et al. 2010). This means that the evolution of friendship and antipathy networks, as well as homophobic attitudes, were simulated whilst taking into account potential interdependencies between friendship and dislike networks, between network and homophobic attitude evolution, and endogenous network effects.

Parameter estimates in the model can be interpreted as the conditional probability of a tie to exist as a function of the explanatory variables, similar to the interpretation of parameter estimates in logistic regression models. Model parameters (explained in the following paragraph) are tested using *t*-ratios, referring to the parameter estimate divided by its standard error. Three RSiena models were estimated, one per school. Conclusions on the hypotheses were based on the combined results. With three schools, a meta-analysis based on means and standard deviations of parameter estimates is of limited use (Ripley et al. 2019). Therefore, a meta-analysis directed at testing parameters that served as tests of the hypotheses was conducted, by means of Fisher-type test for combining independent *p*-values (Fisher 1932). This method produces two one-sided tests: One with an alternative hypothesis that for at least one network the parameter estimate is smaller than zero, and one with an alternative hypothesis that for at least one network the parameter estimate is greater than zero. As two tests are conducted for each parameter, inference with regard to statistical significance was based on *p*-values of *α*/2, with *α* set at 0.05. On a more informal level, consistency in parameters across schools, referring to parameters having the same signs across school networks, delivered the strongest support for hypotheses.

#### Missing data and composition change

Missing network data were handled through the default RSiena procedure called last value carried forward method (Ripley et al. 2019) in which the impact of imputations on the results is minimized (Huisman and Steglich 2008). For each missing tie variable, the non-missing value (if any) is imputed; if the previous values are missing and for missing wave 1 ties, the value 0 (referring to the absence of a tie) is assigned. Whenever imputed values are used, parameter estimate updates are based on the non-imputed parts of the data. Missing covariate data are, by default, replaced by the variable’s global mean.

To account for school composition changes (e.g., participants joining and leaving schools in between the two waves of data collection), the method of Huisman and Snijders (2003) was used. In short, this method tells the simulation algorithm at which point in time between waves which participants join or leave schools. Final year students in School 2 left the school a little less than one month before the second wave of data collection. Other joiners and leavers were assumed to have left or joint schools halfway in between school waves. Friendship and antipathy ties to and from joiners or leavers before they joint or after they left the school were set at zero.

#### Model specification

In order to test hypotheses, integrated friendship-dislike-homophobic attitudes co-evolution models were estimated. By estimating an *average similarity effect* for both friendships and antipathies in the homophobic attitudes dynamics part of the model, it was tested whether or not participants would assimilate with their friends in terms of homophobic attitudes (Hypothesis 1; expecting a positive parameter estimate) or become more dissimilar to their foes in terms of homophobic attitudes (Hypothesis 2(b); expecting a negative parameter estimate). More specifically, the average similarity effects estimate the tendency to grow closer to the average homophobic attitudes of participants’ outgoing ties (for details see Appendix C). It thus captures whether participants are influenced by peers they themselves consider their friends or foes. By estimating the *homophobic attitudes similarity* effect in both the friendship and antipathy dynamics part of the network, it was tested whether friendships (Hypothesis 3; expecting a positive parameter estimate) or antipathies (Hypothesis 4(b); expecting a negative parameter estimate) would be created or maintained more often between individuals with similar (for friendships) or dissimilar (for antipathies) homophobic attitudes. Such similarity effects are typically estimated and interpreted in combination with *ego* (given nominations) and *alter* (received nominations) effects. These *homophobic attitudes ego* and *homophobic attitudes alter* effects show whether more homophobic participants give or receive more nominations as friend and foe. A detailed description of the full model specification is provided in Appendix C.

## Results

### Descriptive Statistics

**Fig. 1:**
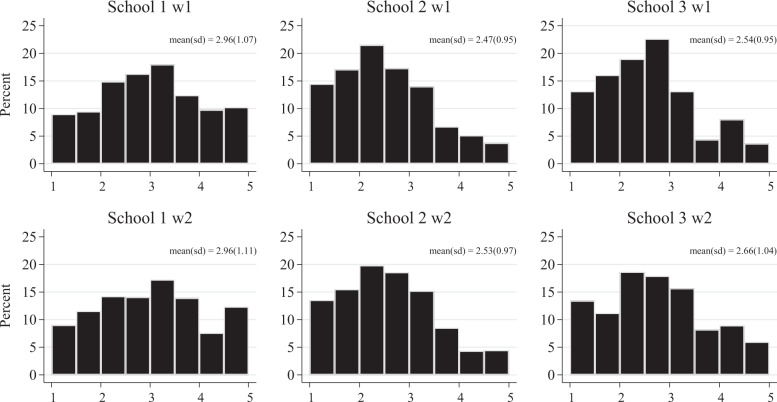
Homophobic attitudes per wave per school. Higher scores reflect more homophobic attitudes

**Fig. 2:**
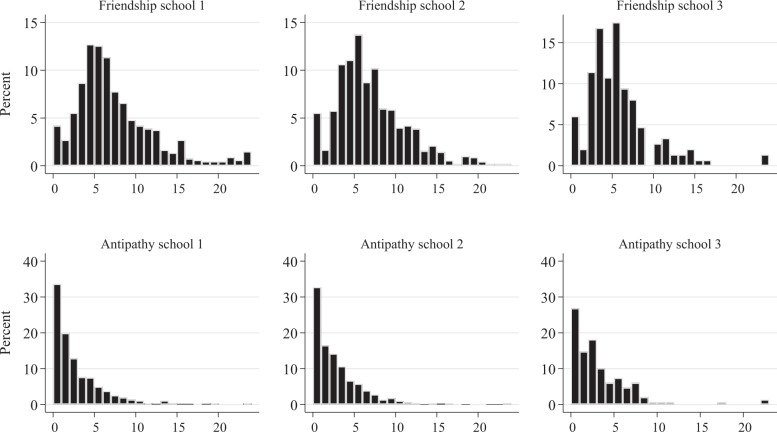
Outdegree distributions friendship and antipathy networks wave 1

Figure 1 plots homophobic attitudes per school and wave. The distribution of attitudes was nearly symmetrical in School 1 and somewhat right-skewed in Schools 2 and 3. On average, participants were somewhat less homophobic in Schools 2 and 3 than in School 1. Table 2 provides descriptive statistics for the friendship networks, antipathy networks, and homophobic attitudes dynamics. Participants nominated between 5.01 (School 3) and 6.96 (School 1) best friends and between 2.91 (School 3) and 2.23 (School 1) foes. 49% of friendship ties concerned participants from different classrooms, and 15% of friendship ties concerned participants from different grades. For antipathy ties, these percentages were 52 and 13 (all calculated using wave 1 data). Figure 2 plots the number of friends and foes participants nominated at wave 1. Most participants indicated to have a few friends, with a small minority indicating to have no (around 5%) or a large number of friends. The figures for the antipathy networks reveal a different pattern. Around 30% of participants did not nominate peers as foes. Of the participants that disliked peers, most nominated less than 5 peers, and only a small minority of participants indicated to dislike a large number of peers.

**Table 2:** Sample and sample change statistics for friendships, antipathies, and homophobic attitudes

	School 1 (*n* = 756)				School 2 (*n* = 1011)				School 3 (*n* = 167)			
	Friendship		Antipathy		Friendship		Antipathy		Friendship		Antipathy	
Sample	Wave 1	Wave 2	Wave 1	Wave 2	Wave 1	Wave 2	Wave 1	Wave 2	Wave 1	Wave 2	Wave 1	Wave 2
Network density indicators	0.008	0.008	0.003	0.003	0.007	0.007	0.003	0.003	0.032	0.030	0.018	0.014
Average degree	6.963	6.599	2.465	2.227	6.566	6.624	2.555	2.953	5.342	5.015	2.906	2.348
Missing fraction	0.115	0.142	0.115	0.142	0.106	0.280	0.106	0.280	0.108	0.192	0.108	0.192
Other network indicators												
Reciprocity (edgewise)	0.410	0.408	0.098	0.058	0.479	0.483	0.100	0.112	0.455	0.470	0.162	0.145
Transitivity	0.259	0.277	0.080	0.078	0.332	0.327	0.076	0.113	0.368	0.369	0.185	0.149
Sample change	Wave 1–Wave 2		Wave 1–Wave 2		Wave 1–Wave 2		Wave 1–Wave 2		Wave 1–Wave 2		Wave 1–Wave 2	
Network changes												
Jaccard index (stability)	0.36		0.15		0.42		0.18		0.42		0.21	
No. of ties dissolved	2026		1125		1717		1157		265		257	
No. of ties emerged	1799		965		1831		1457		257		186	
No. of ties maintained	2146		365		2612		564		381		116	
Changes in homophobic attitude												
No. of steps down	232				194				50			
No. of steps up	281				305				66			
Actors that remain stable	229				285				47			

Reciprocity was calculated as 2*M*/(2*M* + *A*), where *M* = mutual ties and *A* = asymmetric ties. Transitivity was calculated as *N* of transitive triplets divided by *N* of 2-paths (potentially transitive triplets). For more information on the calculation of the different network indices see Veenstra and Steglich (2012)

The Jaccard index is an indicator of network stability and is calculated as the proportion of ties present at both waves compared with ties present at both waves and either one of the waves. The Jaccard index for the friendship networks (0.36–0.42) indicated sufficient stability (Ripley et al. 2019). Stability in the antipathy network was lower (0.15–0.21), as is typically seen in studies on negative networks in adolescence (e.g., Berger and Dijkstra 2013). Between-wave variation in homophobic attitudes was sufficient for modeling attitude dynamics over time: Only about 30% of participants did not move to a different category on the homophobic attitudes scale between waves 1 and 2 (number of “actors that remain stable” in Table 2). The fraction of missing network data by wave was also provided in Table 2. The fraction of missing network data was relatively high for wave 2 in School 2 (28%), because 211 exam class students in that school had finished classes by the time data for wave 2 were collected. For the time-constant covariates summarized in Table 1, it holds that they were constructed using information provided at either wave 1 or wave 2 in order to minimize missing information. Consequently, missingness was low and ranged between 0% (gender in Schools 1 and 3) and 8.4% (sexual orientation School 3).

### RSiena Results

#### Results regarding social influence

Table 3 displays all hypotheses-related coefficients of the RSiena models. Displayed effects were estimated simultaneously, together with a large number of endogenous network mechanisms (see Appendix D) and should thus be interpreted as conditional effects. The model specifications showed adequate to good fit (results available from the first author). Between waves, participants moved closer to their friends in homophobic attitudes as indicated by the positive average similarity effects, which was in line with Hypothesis 1. The Fisher-type combined test indicated that the average similarity effects was positive (*p* = 0.001). In all three school networks, the parameter estimates were positive, with two of the *t*-ratios being statistically significant two-sidedly at *α* = 0.05 (Schools 1 and 3), and the third being significant one-sidedly (School 2). No evidence was found for either a negative (*p* = 0.173) or a positive (*p* = 0.850) average similarity of foes effect in the homophobic attitude dynamics part of the model, thus finding no support for Hypothesis 2, which stated that participants would become more dissimilar in homophobic attitudes compared with their foes.

**Table 3:** Selected results RSiena friendship–antipathy–homophobic attitudes co-evolution analysis

Effect	School 1		School 2		School 3		Fisher < 0	Fisher > 0
	Par.	(*SE*)	Par.	(*SE*)	Par.	(*SE*)		
Homophobic attitudes dynamics								
Average similarity (friendship)	4.359*	(2.118)	2.348ǂ	(1.308)	5.543*	(2.681)	>0.999	0.001
Average similarity (antipathy)	−8.244	(5.020)	0.043	(3.475)	−0.741	(4.685)	0.173	0.850
Friendship dynamics								
Homophobic attitudes alter	0.001	(0.011)	−0.037**	(0.014)	0.028	(0.035)	0.052	0.602
Homophobic attitudes ego	0.019ǂ	(0.011)	−0.020	(0.014)	0.020	(0.033)	0.441	0.181
Homophobic attitudes similarity	−0.096	(0.103)	0.017	(0.154)	−1.156***	(0.348)	0.003	0.924
Antipathy dynamics								
Homophobic attitudes alter	−0.008	(0.018)	0.036*	(0.016)	0.000	(0.038)	0.729	0.092
Homophobic attitudes ego	0.017	(0.011)	−0.025*	(0.013)	−0.024	(0.033)	0.107	0.425
Homophobic attitudes similarity	0.069	(0.127)	−0.045	(0.124)	−0.356	(0.303)	0.322	0.733

All convergence *t*-ratios < |0.07|, overall maximum convergence ratio models all smaller than 0.21

ǂ*p* < 0.10; *p < 0.05; **p < 0.01; ***p < 0.001, two-sided

#### Results regarding selection

No clear pattern in results was detected regarding how similarity in homophobic attitudes was related to friendship dynamics (homophobic attitudes similarity effect). The Fisher-type combined test indicated that there was no significant evidence for positive selection (*p* = 0.924) (Hypothesis 3). If anything, the test pointed to a negative effect (*p* = 0.003). As evident from parameter estimates of individual school networks, this result was driven by a significantly negative estimate in School 3. In the other two schools, the homophobic attitudes similarity effects were not significant, with one parameter estimate being positive and the other being negative.

No evidence was found for either negative (*p* = 0.322) or positive (*p* = 0.733) homophobic attitudes similarity effects in the antipathy dynamics part of the network. It was thus not found that dissimilarity in homophobic attitudes increased the chance of participants disliking one another (Hypothesis 4).

#### Re-specification of the negative influence hypothesis

**Fig. 3:**
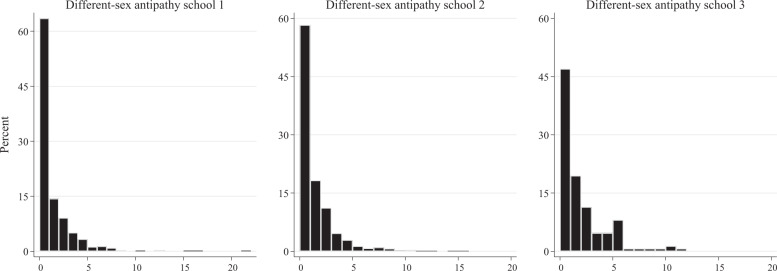
Degree distributions cross-sex antipathy networks wave 1

In order to test whether negative influence and selection would be observed between participants of a different sex, models were rerun, preserving from the antipathy networks only ties between boys and girls. Descriptive information on this constrained antipathy network is provided in Table 4 and Fig. 3. Across schools, participants on average indicated to dislike one peer of a different sex. Figure 3 furthermore indicates that a substantial proportion of participants (roughly between 40 and 55%) reported such cross-sex antipathy nominations. They thus comprise a sizable subpart of participants’ antipathy networks.

**Table 4:** Sample and sample change statistics cross-sex antipathy network

	School 1		School 2		School 3	
Sample	Wave 1	Wave 2	Wave 1	Wave 2	Wave 1	Wave 2
Network density indicators	0.001	0.001	0.001	0.001	0.001	0.012
Average degree	0.967	0.855	1.008	1.238	1.570	1.341
Missing fraction	0.115	0.142	0.106	0.280	0.108	0.192
Other network indicators						
Reciprocity (edgewise)	0.087	0.054	0.088	0.124	0.197	0.155
Transitivity^a^	0.000	0.000	0.000	0.000	0.000	0.000
Sample change	Wave 1–Wave 2		Wave 1–Wave 2		Wave 1–Wave 2	
Jaccard index (stability)	0.15		0.17		0.22	
No. of ties dissolved	457		469		128	
No. of ties emerged	370		624		105	
No. of ties maintained	140		222		67	

^a^Transitive triads are impossible in these networks (a cross-sex foe of your cross-sex foe always has the same sex as you, assuming there are only boys and girls)

Table 5 displays selected results of the RSiena analysis using this constrained antipathy network instead of the original antipathy network. Two things stand out. First, results are in line with a faultline effect in network selection (Hypothesis 4b). The Fisher-type combined test indicated that at least one of the average similarity effects was negative (*p* < 0.001), whilst there was no evidence pointing to positive similarity effects (*p* > 0.999). Looking at the parameter estimates of the different school networks, all effects were negative, with two of the *t*-ratios of estimates for individual schools being statistically significant two-sidedly at *α* = 0.01 (Schools 1) and at *α* = 0.001 (School 3), and the third being not statistically significant, but in the same direction. Only limited evidence was detected for negative influence, however (Hypothesis 2b). Parameter estimates differed in sign across schools, with the Fisher-type combined test returning a non-significant result, although the parameter estimate of the average similarity effect was negative and significant one-sidedly at *α* = 0.05 in one of the school networks (School 1).

**Table 5:** Selected results RSiena friendship–antipathy–homophobic attitudes co-evolution analysis, antipathy ties constrained to cross-sex peers

Effect	School 1		School 2		School 3		Fisher < 0	Fisher > 0
	Par.	(*SE*)	Par.	(*SE*)	Par.	(*SE*)		
Homophobic attitudes dynamics								
Average similarity (friendship)	4.298*	(1.922)	2.369ǂ	(1.280)	7.499	(4.723)	>0.999	0.002
Average similarity (antipathy)	−10.057ǂ	(5.284)	4.024	(3.518)	6.420	(7.378)	0.252	0.278
Friendship dynamics								
Homophobic attitudes alter	0.000	(0.011)	−0.035*	(0.014)	0.025	(0.037)	0.065	0.657
Homophobic attitudes ego	0.017	(0.011)	−0.020	(0.014)	0.033	(0.033)	0.478	0.150
Homophobic attitudes similarity	−0.104	(0.102)	0.023	(0.137)	–1.117**	(0.356)	0.004	0.918
(Constrained) antipathy dynamics								
Homophobic attitudes alter	−0.023	(0.021)	0.059**	(0.020)	–0.010	(0.047)	0.454	0.025
Homophobic attitudes ego	0.017	(0.016)	−0.042*	(0.018)	–0.071ǂ	(0.043)	0.016	0.681
Homophobic attitudes similarity	−0.569**	(0.177)	−0.672***	(0.172)	−0.621	(0.427)	<0.001	>0.999

All convergence *t-*ratios < |0.04|, overall maximum convergence ratio models all smaller than 0.12

ǂ*p* < 0.10; *p < 0.05; **p < 0.01, two-sided

Together, these results indicate that when adolescents differ from their peers in multiple traits (here sex and homophobic attitudes) the likelihood of disliking these peers increases. No evidence was found, however, for adolescents developing more dissimilar homophobic attitudes compared with cross-sex peers they disliked. This respecification did thus not deliver evidence in line with the negative influence hypothesis.

### Sensitivity Analyses

A number of tests were conducted in order to increase the robustness of findings. The results of these additional analyses are available from the first author.

#### Exploring potential conditionality of effects

First, antipathy networks were constrained to take into account actors that were either of different ethnicity or sex, or both. The results were similar to the ones presented.

Second, it was checked whether the overall selection and influence effects regarding homophobic attitudes were conditional on either sex or grade, given the considerable age range of participants and as homophobic attitudes may be a more salient topic among boys than among girls (Slaatten et al. 2014). No systematic differences were detected between boys and girls or between grades regarding hypothesis testing effects.^4^

Third, to better understand the unexpected negative selection effect in School 3, supplementary analyses with the School 3 data were conducted, testing interaction effects between the negative homophobic similarity effects and a range of ego and dyadic effects, in order to find out whether this effect was driven by subgroups of participants in that school. These effects included ego and dyadic effects for sex, age, grade, ethnicity, and popularity. None of these interactions were statistically significant, nor did they lead to a substantial change in the size, direction, or significance of the homophobic attitudes similarity effect.

#### Homophobic attitudes and friendship selection

In response to the inconsistent way in which similarity in homophobic attitudes was related to friendship dynamics, analyses were rerun, this time using a more elaborate specification of how homophobic attitudes might be related to friendship dynamics. This specification followed recent recommendations by Snijders and Lomi (2019) regarding the specification of selection functions on ordinal or continuous attributes in positive social networks, such as homophobic attitudes in friendship networks. Results, discussed in detail in Appendix E, led to a similar conclusion as the original model, namely that homophobic attitudes were not consistently related to friendship selection in the sample.

#### Excluding School 2

It was checked whether conclusions depended on the high amount of composition change in School 2, where exam classes had finished school before the second wave. Importantly, conclusions would not have changed in case the results of School 2 would have been disregarded: The results of Fisher-type tests using Schools 1 and 3 only would have led to the same statistical inferences on results of interest as the inferences presented above.

## Discussion

Previous research suggests that homophobic attitudes are subject to peer influence in adolescence (Poteat 2007), but evidence on specific mechanisms is lacking. Therefore, the social salience of homophobic attitudes in the adolescent peer context was assessed by employing longitudinal social network models and examining the potential influence of both friends and foes. Furthermore, it was studied whether (dis)similarity in homophobic attitudes made adolescents more likely to select peers as friends or foes. Findings indicated that friends grew closer in homophobic attitudes over time, in line with the social influence hypothesis. However, similarity in homophobic attitudes did not increase the likelihood to select peers as friends in a consistent manner. Follow-up analyses indicated that having very different homophobic attitudes increased the likelihood of disliking between peers of different sex, but no evidence was found for adolescents developing more polarized homophobic attitudes compared with the peers they disliked over time.

Study findings were in line with earlier research claiming that peers influence each other’s homophobic attitudes (Poteat et al. 2007; Prati 2012; Slaatten et al. 2015). This study adds to the literature by confirming this finding within a framework with particular methodological rigor. That is, it was shown that adolescents grow closer to the attitudes of their friends over time, suggesting social influence. Following arguments derived from social learning theory, subjective group dynamics, and the social reasoning developmental perspective, it was claimed that this social influence could be a consequence of a strong attunement to the norms within adolescent peer groups. Future studies employing the experimental design common in this literature (Abrams et al. 2003), could elucidate whether attunement to group norms indeed leads to adolescents changing their homophobic attitudes, or whether social influence might be due to other mechanisms, such as the dyadic interactions that the theoretical literature on attitude dynamics argues to be the driving force behind opinion change (Flache et al. 2017).

No consistent evidence was found for adolescents being more likely to select each other as friends when they were more similar in homophobic attitudes. This could mean that homophobic attitudes might not operate as a selection criterion for friendship selection in adolescence. That could imply that the verbalization of attitudes during conversations, usage of homophobic epithets, or display of homophobic behavior, which were argued to facilitate selection processes, might have been less common than assumed. Stronger effects might have been detected when more visible aspects of homophobic prejudice than attitudes would have been measured, such as homophobic bullying or name-calling. It has been found that adolescents do not only display homophobic behavior in order to express homophobic attitudes, but also more instrumentally, for instance to acquire social status or as a bullying strategy (Fulcher 2017). This means that this study provided evidence on influence and selection mechanisms on an attribute that is closely linked to the concept of homophobia. However, having measured homophobic behavior would have given us the opportunity to analyze whether youth update their homophobic attitudes through observing peers that employ homophobic behavior strategically, as was argued during hypothesis building.

In addition, results in one of the schools pointed to a negative effect of similarity in homophobic attitudes on friendship selection. This means that adolescents were less likely to establish or maintain friendship ties with others, the more similar they were in homophobic attitudes. Although this pattern was found in only one school (School 3), it provides room for speculation. As School 3 was by far the smallest school in the study, the negative selection effect found there could suggest that under structural constraints one might be more likely to accept that friends hold different opinions. That is, in social situations in which no or only a few likeminded peers might be available, the desire to acquire and maintain friendships might trump the preference for friends with similar opinions.

No evidence was found for adolescents who disliked each other to become more dissimilar in homophobic attitudes over time, in line with other research that fails to find convincing empirical evidence for negative influence as a mechanism of social influence (for an overview see Flache et al. 2017). Previous research on negative social influence used experiments, whereas this comprised a field study, examining attitude dynamics over a longer period of time, making these findings a valuable extension to this line of work. In addition, results are of interest in light of the intriguing field experiment among Twitter users (Bail et al. 2018), where evidence in line with negative influence *was* detected. Whilst recognizing that Twitter users with opposing political views certainly do not map one-to-one onto disliked peers in adolescence, and whilst cautioning readers against equating non-significant results with a validation of the null hypothesis that there is no negative influence, some speculations of these differences in findings can be offered. The differences could mean that negative influence can be induced in a situation of forced exposure to others with strongly opposing views. This forced contact might for instance play a role within political polarization, which originates in an institutional context where political opponents are in repeated forced contact with one another. In contexts where people have more freedom in whom to interact with, such as for instance in the high school context, they might opt for avoiding or ignoring people that hold opposing views or whom they dislike, rather than being negatively influenced.

Relatedly, the null findings regarding negative influence could indicate that the extent to which adolescents are aware of the homophobic attitudes of their foes might have been overestimated, despite a sizable literature attesting the salience and ubiquity of homophobia in the adolescent peer context (e.g., Collier et al. 2013). As shown in Figure 1, most participants did not have extreme opinions regarding homosexuality. Therefore, they may not have manifested their attitudes as behaviors frequently. Therefore, in such cases youth may make assumptions of the homophobic attitudes of their foes based on stereotypes, which could prevent negative influence in case such assumptions are inaccurate.

An even more pessimistic interpretation of results could be that homophobic attitudes did not comprise a salient topic in the adolescent peer context, and that participants only discussed them after being primed to do so after the first wave of data collection. Consequently, participants might have discovered the homophobic attitudes of their friends, but not of their foes, as foes might have avoided each other’s company. If true, homophobic attitudes may not be a critical factor along which Dutch adolescents align.

This study has some limitations. First, stochastic actor oriented models lack a straightforward interpretation of effects in terms of their effect size (Ripley et al. 2019).^5^ Although some efforts have been made to evaluate results from stochastic actor-oriented models in terms of effect size (Indlekofer and Brandes 2013; Ripley et al. 2019), the interpretability of these measures is limited, as they lack the intuitive appeal of effect size measures from linear models. Consequently, evidence was evaluated primarily in terms of statistical significance, although it must be acknowledged that if possible, *p*-values should not be the sole parameter to look at when evaluating research evidence (McShane et al. 2019). Therefore, caution in interpreting results was applied by performing multiple theoretically informed follow-up analyses, and weighting evidence by considering consistency in findings across schools, in addition to statistical significance only.

Second, the employed measure of homophobic attitudes only included items referring to gay men or homosexuality in general. Items specifically referring to lesbian women were not included. This is common in the field, where more instruments for the measurement of homophobic attitudes have been developed that include no or only a few items explicitly referring to lesbian women (Siebert et al. 2009; van Wijk et al. 2005; Wright et al. 1999). Nonetheless, this choice could have influenced results. As mentioned, gay men are the subgroup within the population of sexual minority individuals that face the most negative prejudice (Swank and Raiz 2010). It could thus be that smaller effects would have been found had items about lesbian women been included in the scale, as feelings about this group might have been less strong and thus less salient.

Lastly, although the analyses controlled for gender, ethnicity and participants’ own sexual orientation, traits that are known to correlate strongly with homophobic attitudes (Costa et al. 2013), a remaining threat to conclusions is that results are consequence of latent homophily. That is, instead of students selecting peers based on or affecting their peers’ homophobic attitudes specifically, selection and influence might have taken place on progressive or conservative sociopolitical stances more generally instead. Unfortunately, as homophobic attitudes were the only sociopolitical attitude measure collected in the data, this is not something that could be explored and is thus left for future work to remedy. A strategy for solving this issue could be to collect information on a number of sociopolitical attitudes in addition to homophobic attitudes and simultaneously estimate selection and influence processes with regard to them. If selection or influence indeed takes place on general conservatism or progressiveness rather than on specific issues, the simultaneous inclusion of multiple attitudes could make the here detected effects disappear as a consequence of multicollinearity.

## Conclusion

Homophobic attitudes and behavior are a widespread problem among adolescents. Nevertheless, what the role of the peer context is in shaping these attitudes is unclear. Therefore, the aim of this study was to investigate the role of friendships and antipathies in the development of homophobic attitudes in adolescence by conducting longitudinal social network analysis. Participants were found to grow closer to the homophobic attitudes of their friends over time. Furthermore, findings from this study add to the growing body of literature that questions the existence of negative social influence. It was found, however, that having very different homophobic attitudes increased the chance for boys and girls to dislike each other. In addition, no evidence for positive selection was found, suggesting that friendship bonds will not be broken over dissimilar levels of homophobic attitudes. Together, these findings suggest that although a homophobic peer climate can spread through friendship networks, tolerant attitudes regarding sexual diversity may spread in the same way.

## Supplementary material

Appendix

## Footnotes

1 The original sample included a fourth school. This school contained a practical education track, in which students spent a substantial proportion of time doing work placements. More than a quarter of students from this school were absent during both waves of the study. This school was excluded, because this exceeds the maximal tolerable proportion of missing data for the employment of longitudinal social network analysis (Huisman and Steglich 2008).

2 https://www.cbs.nl/en-gb/our-services/methods/definitions?tab=m#id=migration-background

3 The Dutch language does not have a direct translation of the word gay. The most standard term for gay, homosexual, or being same-sex attracted would be “homoseksueel” (often shortened to “homo”).

4 Specifically, it was examined whether systematic differences between grades or between boys and girls existed by conducting score-type tests (Ripley et al. 2019). Interaction terms between dummies for grades or sex and selection and influence effects were added to the model and were constrained at zero. Large misfit between the data under the restricted model (assuming no differences in selection and influence effects between grades or sex) and the observed data (allowing for differences in these tendencies between grades or sex) would result in large test statistics, suggesting that no restrictions of (some of the) parameters results in improved model fit.

5 Note for instance that the average similarity effects used to estimate social influence tend to return substantially larger parameter estimates and standard errors than other variables (Ripley et al. 2019), but this should not be interpreted as an indication of their strength.
