# Supplementary material for: Peers and Homophobic Attitudes in Adolescence: Examining Selection and Influence Processes in Friendships and Antipathies
Source: J Youth Adolesc. 2020 Aug 13;49(11):2229–45. doi: 10.1007/s10964-020-01298-8 (PMC7538412; doi:10.1007/s10964-020-01298-8)
Supplement: Supplementary file 1 — Appendix [file 10964_2020_1298_MOESM1_ESM.docx]

**Appendix**

Contents

[Appendix A. Homophobic Attitudes Measure 2](#_Toc47957487)

[Appendix B. Comprehensibility Analysis, Confirmation Psychometric Properties, and Validity Homophobic Attitudes Measure 3](#_Toc47957488)

[Appendix C. Model Specification RSiena Models 11](#_Toc47957489)

[Appendix D. RSiena Results 17](#_Toc47957490)

[Appendix E. Selected Results Respecified Friendship Selection 18](#_Toc47957491)

[References 22](#_Toc47957492)

# Appendix A. Homophobic Attitudes Measure

**Table A1. Homophobic attitudes items used**

| Item | Formulation |
| --- | --- |
| 1. | Homosexual men shouldn’t behave so over the top^1^ |
| 2. | I think homosexuals are disgusting^2^ |
| 3. | I am starting to get tired of all the attention for homosexuals^1^ |
| 4. | Homosexual men are just not real men^3^ |
| 5. | Sex between two men is just plain wrong^3^ |
| 6. | I think that all the attention for homosexuality is getting annoying^1^ |
| 7. | Homosexual men put too much emphasis on their sexual orientation^1^ |
| 8. | I find it disgusting when two men kiss^4^ |

Note: Scale items were obtained/adapted from the following sources:

^1^ van Wijk, E., van De Meerendonk, B., Bakker, F., & Vanwesenbeeck, I (2005). Moderne homonegativiteit: De constructie van een meetinstrument voor het meten van hedendaagse reacties op zichtbare homoseksualiteit in Nederland [Modern homonegativity: The construction of a measurement instrument for measuring current day reactions to visible homosexuality in the Netherlands].” *Tijdschrift Voor Seksuologie* 29, 19–27.

^2^ Herek, G. M. (1988). Heterosexuals’attitudes toward lesbians and gay men: Correlates and gender differences. *The Journal of Sex Research*, *25*(4), 451–477. <https://doi.org/10.1080/00224498809551476>

^3^ van de Meerendonk, B., Eisinga, R., & Felling, A. (2003). Application of Herek’s attitudes toward lesbians and gay men scale in the Netherlands. *Psychological Reports*, *93*(1), 265–275. <https://doi.org/10.2466/pr0.2003.93.1.265>

^4^ Kuyper, L. (2015). *Jongeren en seksuele oriëntatie. Ervaringen van en opvattingen over homoseksuele, biseksuele en heteroseksuele jongeren [Youth and sexual orientation. Experiences of and attitudes towards homosexual, bisexual, and heterosexual youth]*. Den Haag, the Netherlands: Netherlands Institute for Social Research/SCP.

# Appendix B. Comprehensibility Analysis, Confirmation Psychometric Properties, and Validity Homophobic Attitudes Measure

In addition to the data presented in the manuscript, a second data collection was conducted in order to 1) demonstrate that the items used in the scale were comprehensible for young respondents, 2) confirm the psychometric properties of the scale, and 3) ascertain its validity. To this end, below we present evidence collected as part of a study on Dutch youth’s sexual diversity, as well as their attitudes towards sexual diversity.

**Sample**

Data was collected in May and June 2017 from students in 53 classes from 6 Dutch high schools. Schools were located in the north and middle part of the country, in both rural areas and midsized Dutch cities. Students filled in the pen and paper questionnaire within one school hour. Classrooms were arranged in an exam seating arrangement at the time of data collection, with at least one research assistant present during data collection to oversee the data collection process.

Due to the young age of the respondents and sensitive nature of the topic, an active parental consent procedure was sought: students in participating classes were only asked to fill in a questionnaire in case parents approved them to be approached for participation, and only consenting students filled in the questionnaire. The study received IRB approval from the department of Sociology at the University of Groningen*.* Six hundred (600) respondents filled in the questionnaire. Several checks were performed to identify and remove respondents with dubious response patterns from the data. This included eye balling, evaluating open response categories, split half correlations of items coming from the same scale, checks for monotone response patterns, and using Mahalanobis distance techniques for identifying unusual response patterns. This led to the removal of 10 respondents from the sample, resulting in a final sample of 590 respondents.

Table B1 provides sample descriptives. Respondents were 12 to 17 years of age, and the sample was fairly evenly distributed with regard to the sex of respondents. Most respondents and parents were born in the Netherlands. Approximately 58 % of respondent was in a higher educational track.

Table B1. Sample descriptives

|  | Mean (SD) /  Percentage (n) | Missing % (n) |
| --- | --- | --- |
| Age (12 – 17) | 14.04 (1.33) | 1.4 % (8) |
| Sex |  | 1.5 % (9) |
| Girl | 46.6 % (271) |  |
| Boy | 53.4 % (310) |  |
| Country of birth child |  | 0.9 % (5) |
| Netherlands | 97.1 % (568) |  |
| Other country | 2.9 % (17) |  |
| Country of birth father |  | 1.0 % (6) |
| Netherlands | 94.96 % (554) |  |
| Other country | 5.1 % (32) |  |
| Country of birth mother |  | 0.9 % (5) |
| Netherlands | 94.5 % (553) |  |
| Other country | 5.5 % (30) |  |
| Grade and track |  | 0.0 % (0) |
| Grade 7, lower track | 11.5 % (68) |  |
| Grade 7, higher tracks | 14.2 % (84) |  |
| Grade 8, lower track | 13.1 % (77) |  |
| Grade 8, higher tracks | 16.1 % (95) |  |
| Grade 9, lower track | 17.5 % (103) |  |
| Grade 9, higher tracks | 12.5 % (74) |  |
| Grade 10, lower track |  |  |
| Grade 10, higher tracks | 12.47 % (73) |  |
| Grade 11, lower track |  |  |
| Grade 11, higher tracks | 2.7 % (16) |  |

**B1. Comprehensibility of scale items**

The scale used in the original paper consisted of items from existing scales used for the measurement of homophobic attitudes that we deemed comprehensible for even our youngest respondents. To proof this, we asked respondents to rate all items of all original scales in the original 5-point scale (1 = *Strongly agree* to 5 = *Strongly disagree*). In addition, respondents could indicate as a sixth response option that they did not understand the question. As is depicted in Figure B1 below, almost all respondents comprehended items 1-5 and items 7 and 8. A small proportion of respondents (*n* = 56 / 9.4% of the sample) indicated to not comprehend item 6 of the scale (“I think that all the attention for homosexuality is getting annoying”). However, the correlation between a scale using all items and a scale excluding item 6 was almost one (*r*=.996). Furthermore, using a scale excluding item six as the measurement of homophobic attitudes in our RSiena model returned very similar results compared to models using all eight items for measuring homophobic attitudes (results available from the first author). This led us to use all 8 items for measuring homophobic attitudes.

Note that the response pattern in Figure B1 is not a consequence of respondents not wanting to admit to not comprehend an item. As a proof of this, Figure B2 depicts the number of respondents indicating to not know what items meant for all the original scales from which items were extracted in this study: the Attitudes Towards Lesbians and Gays (ATLG) scale (Herek 1988), an item used by Kuyper (2015), items used by van de Meerendonk et al. (2003) that were not found in the ATLG (that paper used both original items and items derived from the ATLG), and the modern homonegativity scale (van Wijk et al. 2005). As can be seen in Figure B2, numerous items were indicated to not be understood by a substantial number of respondents. This was especially the case for the ATLG. For six of the items from that scale, more than 10 percent of respondents indicated not to comprehend the item.

Figure B1. Proportion number of respondents indicating to not understand items used to measure homophobic attitudes

Note: Vertical line is drawn at n=59, reflecting 10 percent of the sample, added as benchmark

Figure B2. Proportion number of respondents indicating to not understand all items measuring homophobic attitudes

Notes: Vertical line is drawn at n=59, reflecting 10 percent of the sample, added as benchmark. Graph titles depict item number of item used in scale in study and Table A1.

**B2. Confirmation of psychometric properties**

In addition to proving the comprehensability of the scale items, this data collection gave us the opportunity to confirm the psychometric properties of the scale. As was the case in the main study, reliability of the scale was high (α=.92). Because the original scales from which the items were extracted were also measured, we could examine whether the items all loaded strongly on the same latent factor. To this end, we performed an exploratory factor analysis. In order to limit dropout, we included only items that more than 90 % of respondents comprehended in the analysis^[[1]](#footnote-1)^. As the original items came from three different scales and one independent single item, maximally four factors were retained. As can be seen in the screeplot depicted in Figure B3, results point to a 1-factor solution. In addition, Table B2 shows that all items used in our scale loaded strongly on the first latent factor.

Figure B3. Screeplot of factor analysis on all homophobic attitudes items

Table B2. Factor loadings principal factor analysis

| Item | Factor 1 | Factor 2 | Factor 3 | Factor 4 | Uniqueness |
| --- | --- | --- | --- | --- | --- |
| ATLG item 1 | .62 |  |  |  | .49 |
| ATLG item 2 (recoded) |  |  |  |  | .84 |
| ATLG item 3 | .64 |  |  |  | .44 |
| ATLG item 6 | .66 |  |  |  | .49 |
| ATLG item 7 |  |  |  |  | .91 |
| ATLG item 10 | .58 |  |  |  | .53 |
| ATLG item 11 (recoded) | .56 |  |  |  | .65 |
| ATLG item 12 **(item #2)** | .79 |  |  |  | .32 |
| ATLG item 13 | .74 |  |  |  | .39 |
| ATLG item 16 | .78 |  |  |  | .34 |
| ATLG item 17 (recoded) | .52 |  |  |  | .70 |
| ATLG item 18 | .74 |  |  |  | .40 |
| ATLG item 19 | .69 |  |  |  | .42 |
| ATLG item 20 (recoded) |  |  |  |  | .77 |
| item SCP **(item #8)** | .76 |  |  |  | .37 |
| SOCON item 1 (recoded) | .76 |  |  |  | .36 |
| SOCON item 2 **(item #4)** | .80 |  |  |  | .33 |
| SOCON item 3 **(item #5)** | .83 |  |  |  | .28 |
| Mod. Hom. Item 1 |  |  |  |  | .59 |
| Mod. Hom. Item 2 **(item #1)** | .75 |  |  |  | .34 |
| Mod. Hom. Item 3 | .66 |  |  |  | .36 |
| Mod. Hom. Item 4 **(item #3)** | .69 |  |  |  | .29 |
| Mod. Hom. Item 5 **(item #6)** | .76 |  |  |  | .28 |
| Mod. Hom. Item 6 **(item #7)** | .70 |  |  |  | .26 |
| Mod. Hom. Item 7 | .53 |  |  |  | .61 |
| Mod. Hom. Item 8 | .70 |  |  |  | .46 |

Notes: *N* = 379. Only factor loadings of at least .50 in absolute size are displayed.

**B3. Validity**

Lastly, the data presented here provided us the opportunity to demonstrate the validity of our homophobic attitudes scale. Three types of validity were tested. Criterion (concurrent) validity (referring to the extent to which a measure is related to an outcome) was assessed by correlating the scale with a measure of adolescent homophobic behavior. Homophobic behavior was measured with a 5 item subscale of the Homophobic Content Agent Scale, which measured the extent to which adolescents use homophobic epithets (Poteat and Espelage 2005).

Convergent validity (referring to the degree to which to factors that theoretically should be related, are related) was demonstrated by correlating the scale with scales that have been used to measure other socio-political attitudes in adolescence: Gender role attitudes (Galambos et al. 1985), xenophobia towards immigrants (van Zalk et al. 2013), and feelings towards Moroccans (one of the largest non-Western immigrant groups in the Netherlands^[[2]](#footnote-2)^) by means of a ’feeling thermometer’ (see Velasco González et al. 2008).

As can be seen in Table B3, the results confirm criterion and convergent validity of the employed scale. Its correlation with homophobic behavior was high (*r*=.50), as was the correlation with gender role attitudes and xenophobia (*r*=.60, *r*=.57). The correlation with feelings towards Moroccans was somewhat lower, yet still substantial and in the expected direction (*r*=.35).

Lastly, as a proof of the scale’s discriminative validity, we tested whether boys and girls reported different levels of homophobic attitudes, in line with earlier studies finding that men are more homophobic than women (e.g., Costa et al. 2013). In line with expectations, boys (*M* = 2.77, *SD* = 0.91) reported substantially more homophobic attitudes than girls (*M* = 1.93, *SD* = 0.70) in our sample, *t*(579) = 12.33, *p* < .001.

Table B3. Pairwise correlations homophobic attitudes and related concepts for assessing criterion and convergent validity

|  | 1. | 2. | 3. | 4. | 5. |
| --- | --- | --- | --- | --- | --- |
| 1. Homophobic attitudes | - |  |  |  |  |
| 2. Homophobic behavior | .50 | - |  |  |  |
| 3. Gender role attitudes | .60 | .40 | - |  |  |
| 4. Xenophobia | .57 | .36 | .44 | - |  |
| 5. Feelings towards Moroccans | .35 | .27 | .34 | .59 | - |

# Appendix C. Model Specification RSiena Models

Definition RSiena model

An RSiena model consists of a selection of ‘effects’ that specify different functions of the model. We specified a rate function and an evaluation function, which were defined specifically for homophobic attitudes, friendship networks, and antipathy networks. The rate function models the speed by which each network actor gets an opportunity for changing her friendship network, antipathy network, or homophobic attitudes. As is common in the field, the models presented in this manuscript included a constant rate function (‘rate parameter’ in Table D1), without additional rate function effects. The evaluation function, sometimes referred to as ‘objective function’, is the primary determinant of the probabilities of changes in networks or behavior. Probabilities are higher for moving towards states with a higher value of the evaluation function. The evaluation functions are constituted by a weighted sum of effects that define the characteristics of the network and behavior that determine the probabilities of changes (Ripley et al. 2019). As in most RSiena applications, the evaluation function was the main focus of model selection in this study.

Below we extensively discuss the effects included in the final model specification of the evaluation function, an excerpt of which is presented in Table 1 of the manuscript. A summary of the formulas of all effect types included for modelling the evaluation function is provided in Table C1. The results of the full model specification are provided in Table D1.

Model specification

Our final model specification included the following hypothesis-related effects, control effects, and effects included in view of model convergence and goodness-of-fit with regard to auxiliary statistics.

*Hypothesis-related effects.* To test our hypotheses, we estimated integrated friendship-dislike-homophobic attitudes co-evolution models. The following model parameters were included in the models as a test of our hypotheses: By estimating the *homophobic attitudes similarity* effect in both the friendship and antipathy dynamics part of the network, we tested whether friendships (H3, expecting a positive parameter estimate) or disliking (H4, expecting a negative parameter estimate) tend to occur more often between individuals with similar or dissimilar homophobic attitudes. Such similarity effects are typically estimated and interpreted in combination with *ego* (given nominations) and *alter* (received nominations) effects (Steglich et al. 2010). These *homophobic attitudes ego* and *homophobic attitudes alter* effects show whether more homophobic respondents give and/or receive more nominations as friend and antipathy. By estimating an *average similarity effect* for both friendships and antipathies in the homophobic attitudes dynamics part of the model, we tested whether or not respondents were likely to assimilate with their friends in terms of homophobic attitudes (H1, expecting a positive parameter estimate) or become more dissimilar to their antipathies in terms of homophobic attitudes (H2(b), expecting a negative parameter estimate).

***Control effects.*** *Ego, alter*, and *similarity* effects for gender and sexual orientation were included in both the friendship and antipathy dynamics parts of the model to control for network selection based on gender and sexual orientation. In the friendship network we additionally included ego, alter, and similarity effects for ethnicity. In the antipathy dynamics part of the model only the similarity effect for ethnicity was included, as including ego and alter effects led to convergence problems. In addition, *same* school grade and school class effects were included in order to control for friendship selection due to physical proximity within the school context. Only the grade effect was included in the antipathy dynamics part of the network, as the school class effect was not significant and led to convergence issues in one of the networks. Furthermore, direct effects of *gender, ethnicity, and same-sex attraction* on homophobic attitudes were estimated in order to control for the effect of gender, ethnic minority status, and sexual minority status on the level and development of homophobic attitudes.

***Model convergence effects and goodness-of-fit effects.*** *Outdegree(density)* and *reciprocity* effects were by default included in both the friendship and antipathy dynamics part of the model. *Outdegree (density)* reflects the tendency of individuals to nominate others. *Reciprocity* reflects the tendency to reciprocate received nominations.

Two effects were included in the friendship dynamics part of the model only. *Three-cycles* represent cycles of generalized reciprocity (referring to student A nominates student B, student B nominates student C, and student C nominates student A). Furthermore, including the *Geometrically Weighted Edgewise Shared Partners “Forward-Forward”* (*gwespFF*) effect, which represents an alternative expression for transitivity, improved model fit.

As recommended (Ripley et al. 2019), the *indegree popularity, outdegree popularity, and outdegree activity* effects were included in the friendship dynamics part of the network*. Indegree popularity* reflects tendencies for actors with high indegrees (referring to receiving friendship or antipathy nominations) to attract extra incoming ties ‘because’ of their high current indegrees. *Outdegree popularity* reflects tendencies for individuals with high outdegrees (referring to sending friendship or antipathy nominations) to receive extra incoming ties. *Outdegree activity* refers to the tendency for actors with high outdegrees to send out extra outgoing ties due to their high current outdegrees.

We used slightly different effects in the antipathy dynamics part of the network for reasons of model fit (see also Appendix F). In addition to the *indegree popularity* and *outdegree activity* effects, we included the *outdegree truncation* effect. This effect reflects the tendency of individuals to send no antipathy ties. Furthermore, the *transitive triplets* effect was included in the antipathy dynamics part of the network. It reflects a tendency for network closure through transitivity. Although a positive effect of transitivity effect for antipathy dynamics was found in Schools 1 and 2 (‘the enemies of your enemies are your enemies’), whereas a negative effect was expected ex ante (‘the enemies of your enemies are your friends’) based on balance theory argumentation (Heider 1946), the effect was retained in the model specification as its inclusion appeared beneficial for model fit and the theoretical focus of the paper lied on the dyadic, rather than the triadic level.

In addition, we included four cross-network effects, where antipathy dynamics were modelled dependent on some characteristics of the friendship network. As the antipathy relationships were comparatively less stable than the friendship relationships, the inclusion of these effects was likely to be beneficial for the convergence and reliability of estimates of the antipathy dynamics (Snijders et al. 2013). Because SIENA is a co-evolution model, however, effects where friendship dynamics were modelled dependent on analogous characteristics of the antipathy network were included in its simulation (although these estimates are not reported) (Ripley et al. 2019).

There was a slight overlap between the friendship and dislike networks. In total, respondents nominated a peer as both a friend and an antipathy 122 times, which was equal to 1.4% of all outgoing friendship nominations and 2.9% percent of all outgoing dislike nominations. Therefore, we included two effects that modelled this potential overlap in friendship and dislike dyads. The first effect, *from friendship to dislike,* models whether sending a friendship tie is related to the tendency to send a dislike tie to the same peer. The second effect, *antipathy reciprocity with friendship*, modelled whether sending a friendship tie relates to the propensity to receive an antipathy nomination from that same peer. Two more effects were included to further model potential dependencies between friendships and antipathies. *Friendship outdegree (sqrt) to antipathy activity* represents the extent to which actors that nominate many peers as friends will make more choices in the antipathy network. *Friendship to agreement* reflected the tendency for actors who are friends to have outgoing antipathy ties to the same actors.

Lastly, three effects were by default included in the homophobic attitudes dynamics part of the model for reasons of model convergence. The *rate parameter* indicated the rate of change in respondents’ homophobic attitudes between time points. The overall mean and variance of homophobic attitudes were controlled for by the *linear shape* (overall tendency) and *quadratic shape* (a negative parameter indicates regression to the mean, whereas a positive parameter indicates polarization) parameters.

**Missing data and composition change**

Missing network data were handled through the default RSiena procedure called last value carried forward method (Ripley et al. 2019) in which the impact of imputations on the results is minimized (Huisman and Steglich 2008). For each missing tie variable, the non-missing value (if any) is imputed; if the previous values are missing as well, the value 0 (referring to the absence of a tie) is assigned. Whenever imputed values are used, parameter estimate updates are based on the non-imputed parts of the data. Missing covariate data are, by default, replaced by the variable’s global mean. Please note that missing data compensation by for instance multiple imputation was not a possibility. Whilst promising first attempts towards developing an imputation method for longitudinal network data have recently been suggested (Krause et al. 2018), extensions of this method to the multiple network models employed in this study were at the time of writing this article still work in progress.

To account for school composition changes (e.g., participants joining and leaving schools in between the two waves of data collection), we employed the method of Huisman and Snijders (2003). In short, this method tells the simulation algorithm at which point in time between waves which respondents join or leave schools. Final year students in School 2 left the school a little less than one month before the second wave of data collection. Other joiners and leavers were assumed to have left or joint schools halfway in between school waves. Friendship and antipathy ties to and from joiners or leavers before they joint or after they left the school were set at zero.

Table C1. Formulas for effect types for model specification of evaluation function

| Effect | Formula |
| --- | --- |
| *Univariate network dynamics:* |  |
| Outdegree (density) | $\boldsymbol{s}\left( \boldsymbol{x} \right)\boldsymbol{=}\sum_{\boldsymbol{j}} \boldsymbol{x}_{\boldsymbol{ij}}$ |
| Reciprocity | $\boldsymbol{s}\left( \boldsymbol{x} \right)\boldsymbol{=}\sum_{\boldsymbol{j}} \boldsymbol{x}_{\boldsymbol{ij}}\boldsymbol{x}_{\boldsymbol{ji}}$ |
| 3-cycles | $\boldsymbol{s}\left( \boldsymbol{x} \right)\boldsymbol{=}\sum_{\boldsymbol{j,h}} \boldsymbol{x}_{\boldsymbol{ij}}\boldsymbol{x}_{\boldsymbol{jh}}\boldsymbol{x}_{\boldsymbol{hi}}$ |
| GWESPFF (.69)^1^ | $\boldsymbol{s}\left( \boldsymbol{x} \right)\boldsymbol{=}\sum_{\boldsymbol{k=1}}^{\boldsymbol{n-2}} \boldsymbol{x}_{\boldsymbol{ij}} \boldsymbol{e}^{\boldsymbol{\alpha}}\boldsymbol{\{}\boldsymbol{1-(1-}{\boldsymbol{e}^{\boldsymbol{-\alpha}}\boldsymbol{)}}^{\sum_{\boldsymbol{h=1}}^{\boldsymbol{n}} \boldsymbol{x}_{\boldsymbol{ih}}\boldsymbol{x}_{\boldsymbol{hj}}}\boldsymbol{\}}$ |
| Indegree – popularity | $\boldsymbol{s}\left( \boldsymbol{x} \right)\boldsymbol{=}\sum_{\boldsymbol{j}} \boldsymbol{x}_{\boldsymbol{ij}}\sum_{\boldsymbol{h}} \boldsymbol{x}_{\boldsymbol{hj}}$ |
| Outdegree-popularity | $\boldsymbol{s}\left( \boldsymbol{x} \right)\boldsymbol{=}\sum_{\boldsymbol{j}} \boldsymbol{x}_{\boldsymbol{ij}}\sum_{\boldsymbol{h}} \boldsymbol{x}_{\boldsymbol{jh}}$ |
| Outdegree-activity | $\boldsymbol{s}\left( \boldsymbol{x} \right)\boldsymbol{=}\boldsymbol{x}_{\boldsymbol{i+}}^{\boldsymbol{2}}$ |
| Covariate alter | $\boldsymbol{s}\left( \boldsymbol{x} \right)\boldsymbol{=}\sum_{\boldsymbol{j}} \boldsymbol{x}_{\boldsymbol{ij}}\boldsymbol{v}_{\boldsymbol{j}}$ |
| Covariate ego | $\boldsymbol{s}\left( \boldsymbol{x} \right)\boldsymbol{=}\sum_{\boldsymbol{j}} \boldsymbol{v}_{\boldsymbol{j}}\boldsymbol{x}_{\boldsymbol{i+}}$ |
| Same covariate | $\boldsymbol{s}\left( \boldsymbol{x} \right)\boldsymbol{=}\sum_{\boldsymbol{j}} \boldsymbol{x}_{\boldsymbol{ij}}\boldsymbol{I\{}\boldsymbol{v}_{\boldsymbol{i}}\boldsymbol{=}\boldsymbol{v}_{\boldsymbol{j}}\boldsymbol{\}}$ |
| Covariate similarity^2^ | $\boldsymbol{s}\left( \boldsymbol{x} \right)\boldsymbol{=}\sum_{\boldsymbol{j}} \boldsymbol{x}_{\boldsymbol{ij}}\boldsymbol{(}\boldsymbol{sim}_{\boldsymbol{ij}}^{\boldsymbol{v}}\boldsymbol{-}\hat{\boldsymbol{sim}^{\boldsymbol{v}}}\boldsymbol{\}}$ |
| Transitive triplets | $\boldsymbol{s}\left( \boldsymbol{x} \right)\boldsymbol{=}\sum_{\boldsymbol{j,h}} \boldsymbol{x}_{\boldsymbol{ij}}\boldsymbol{x}_{\boldsymbol{ih}}\boldsymbol{x}_{\boldsymbol{hj}}$ |
| Outdegree-truncation | $\boldsymbol{s}\left( \boldsymbol{x} \right)\boldsymbol{=}\min\boldsymbol{(}\boldsymbol{x}_{\boldsymbol{i+}}\boldsymbol{, 1)}$ |
|  |  |
| *Multivariate network dynamics:* |  |
|  |  |
| From friendship to dislike | $\boldsymbol{s}\left( \boldsymbol{x} \right)\boldsymbol{=}\sum_{\boldsymbol{j}} \boldsymbol{x}_{\boldsymbol{ij}}\boldsymbol{w}_{\boldsymbol{ij}}$ |
| Antipathy reciprocity with friendship | $\boldsymbol{s}\left( \boldsymbol{x} \right)\boldsymbol{=}\sum_{\boldsymbol{j}} \boldsymbol{x}_{\boldsymbol{ij}}\boldsymbol{w}_{\boldsymbol{ji}}$ |
| Outdegree (sqrt) friendship activity | $\boldsymbol{s}\left( \boldsymbol{x} \right)\boldsymbol{=}\sum_{\boldsymbol{j}} \boldsymbol{x}_{\boldsymbol{ij}}\boldsymbol{(}\sqrt{\boldsymbol{w}_{\boldsymbol{j+}}}\boldsymbol{-}\sqrt{\bar{\boldsymbol{w}}}\boldsymbol{)}$ |
| Friendship to agreement | $\boldsymbol{s}\left( \boldsymbol{x} \right)\boldsymbol{=}\sum_{\boldsymbol{j\neq h}} \boldsymbol{x}_{\boldsymbol{ij}}\boldsymbol{w}_{\boldsymbol{ih}}\boldsymbol{x}_{\boldsymbol{hj}}$ |
|  |  |
| *Homophobic attitudes dynamics:* |  |
| Linear shape | $\boldsymbol{s}\left( \boldsymbol{z} \right)\boldsymbol{=}\boldsymbol{z}_{\boldsymbol{i}}$ |
| Quadratic shape | $\boldsymbol{s}\left( \boldsymbol{z} \right)\boldsymbol{=}\boldsymbol{z}_{\boldsymbol{i}}^{\boldsymbol{2}}$ |
| Average similarity (friendship or antipathy) | $\boldsymbol{s}\left( \boldsymbol{z} \right)\boldsymbol{=}\boldsymbol{x}_{\boldsymbol{i+}}^{\boldsymbol{-1}}\sum_{\boldsymbol{j}} \boldsymbol{x}_{\boldsymbol{ij}}\boldsymbol{(}\boldsymbol{sim}_{\boldsymbol{ij}}^{\boldsymbol{z}}\boldsymbol{-}\hat{\boldsymbol{sim}^{\boldsymbol{z}}}\boldsymbol{\}}$ |
| Effect from attribute | $\boldsymbol{s}\left( \boldsymbol{z} \right)\boldsymbol{=}\boldsymbol{z}_{\boldsymbol{i}} \boldsymbol{v}_{\boldsymbol{i}}$ |

Notes. Network dynamics are denoted as s(x), attitude dynamics denoted as s(z). A tie from actor i to actor j is called x_ij_. The variables x_i+_ and w_j+_ refer to all outgoing ties of actor i and j, respectively. Variable $\boldsymbol{v}_{\boldsymbol{i}}$ denotes the covariate of actor i. The indicator function *I* equals 1 if the sender and recipient of a tie are of the same Covariate and 0 otherwise. In the multivariate network effects, $\boldsymbol{x}_{\boldsymbol{ij}}$ refers to a tie in the dependent network, whereas $\boldsymbol{w}_{\boldsymbol{ij}}$ refers to a tie in the network that serves as an explanatory variable. All Covariates are centered. $\boldsymbol{z}_{\boldsymbol{i}}$ here refers to the level of homophobic attitudes of actor i.

^1^: (.69) means here that α, a tuning parameter, is set to .69

^2^: $\hat{\boldsymbol{sim}^{\boldsymbol{v}}}$ is the mean of all similarity scores, which are defined as $\boldsymbol{sim}_{\boldsymbol{ij}}^{\boldsymbol{v}}$ = (∆−|vi−vj |)/∆, with ∆ = max_ij_ |vi − vj | being the observed range of the covariate. An analogous definition holds for the similarity terms in the average similarity effect.

# Appendix D. RSiena Results

Table D1. Results complete RSiena models

|  | School 1 |  | School 2 |  | School 3 |  |
| --- | --- | --- | --- | --- | --- | --- |
| Effects | parameter | (*SE*) | parameter | (*SE*) | parameter | (*SE*) |
| *Friendship dynamics:* |  |  |  |  |  |  |
| rate parameter friendship | 22.693 | (0.720) | 27.622 | (1.109) | 11.091 | (0.953) |
| outdegree (density) | –3.716^∗∗∗^ | (0.073) | –3.550^∗∗∗^ | (0.085) | –3.440^∗∗∗^ | (0.262) |
| reciprocity | 1.787^∗∗∗^ | (0.072) | 2.295^∗∗∗^ | (0.070) | 1.718^∗∗∗^ | (0.187) |
| 3-cycles | –0.182^∗∗∗^ | (0.027) | –0.223^∗∗∗^ | (0.031) | –0.197^∗^ | (0.081) |
| GWESPFF (.69) | 1.631^∗∗∗^ | (0.041) | 1.787^∗∗∗^ | (0.043) | 1.399^∗∗∗^ | (0.131) |
| indegree - popularity | –0.024^∗∗∗^ | (0.007) | –0.029^∗∗∗^ | (0.008) | –0.037 | (0.025) |
| outdegree - popularity | –0.045^∗∗∗^ | (0.006) | –0.078^∗∗∗^ | (0.008) | –0.059^∗^ | (0.023) |
| outdegree-truncation | –1.350^∗∗∗^ | (0.340) | –0.034 | (0.428) | –1.177^ǂ^ | (0.624) |
| gender alter | –0.089^∗^ | (0.044) | –0.221^∗∗∗^ | (0.046) | 0.273^∗^ | (0.130) |
| gender ego | 0.170^∗∗∗^ | (0.043) | –0.011 | (0.050) | 0.205 | (0.131) |
| same gender | 0.305^∗∗∗^ | (0.036) | 0.350^∗∗∗^ | (0.042) | 0.685^∗∗∗^ | (0.114) |
| same grade | 0.633^∗∗∗^ | (0.039) | 0.641^∗∗∗^ | (0.038) | 0.542^∗∗∗^ | (0.122) |
| same class | 0.718^∗∗∗^ | (0.036) | 0.406^∗∗∗^ | (0.036) | 0.628^∗∗∗^ | (0.119) |
| ethnicity alter | 0.265^∗∗∗^ | (0.040) | 0.189^∗∗^ | (0.060) | –0.262 | (0.346) |
| ethnicity ego | 0.223^∗∗∗^ | (0.040) | 0.156^∗^ | (0.062) | –1.298^∗^ | (0.556) |
| same ethnicity | 0.308^∗∗∗^ | (0.036) | 0.114^∗^ | (0.055) | –0.289 | (0.305) |
| sexual minority alter | 0.065 | (0.098) | 0.172^ǂ^ | (0.092) | 0.477 | (0.309) |
| sexual minority ego | 0.147 | (0.095) | 0.039 | (0.105) | 0.816^∗^ | (0.373) |
| same sexual orientation | 0.079 | (0.055) | 0.126^∗^ | (0.064) | 0.464^∗^ | (0.207) |
| homophobic attitudes alter | 0.001 | (0.011) | –0.037^∗∗^ | (0.014) | 0.028 | (0.035) |
| homophobic attitudes ego | 0.019^ǂ^ | (0.011) | –0.020 | (0.014) | 0.020 | (0.033) |
| homophobic attitudes similarity | –0.096 | (0.103) | 0.017 | (0.154) | –1.156^∗∗∗^ | (0.348) |
| *Antipathy dynamics:* |  |  |  |  |  |  |
| rate parameter dislike | 18.860 | (0.860) | 22.777 | (1.023) | 16.625 | (1.640) |
| outdegree (density) | –4.480^∗∗∗^ | (0.130) | –4.694^∗∗∗^ | (0.118) | –4.184^∗∗∗^ | (0.300) |
| reciprocity | 0.740^∗∗∗^ | (0.153) | 1.070^∗∗∗^ | (0.094) | 0.920^∗∗∗^ | (0.176) |
| transitive triplets | 0.200^∗∗^ | (0.067) | 0.221^∗∗∗^ | (0.046) | –0.057 | (0.082) |
| indegree - popularity | 0.046^∗^ | (0.023) | 0.033^∗∗^ | (0.010) | 0.073^∗∗^ | (0.028) |
| outdegree - activity | 0.026^∗∗∗^ | (0.003) | 0.022^∗∗∗^ | (0.003) | 0.050^∗∗∗^ | (0.008) |
| outdegree-truncation | –3.103^∗∗∗^ | (0.177) | –2.762^∗∗∗^ | (0.201) | –2.748^∗∗∗^ | (0.361) |
| gender alter | 0.041 | (0.056) | –0.016 | (0.047) | –0.009 | (0.116) |
| gender ego | 0.049 | (0.048) | –0.039 | (0.042) | 0.030 | (0.112) |
| same gender | 0.266^∗∗∗^ | (0.046) | 0.122^∗∗^ | (0.037) | –0.120 | (0.104) |
| same grade | 1.547^∗∗∗^ | (0.068) | 2.043^∗∗∗^ | (0.080) | 1.663^∗∗∗^ | (0.171) |
| same ethnicity | 0.015 | (0.043) | –0.098^∗^ | (0.039) | 0.297^∗^ | (0.134) |
| sexual minority alter | 0.298^∗∗^ | (0.112) | 0.049 | (0.097) | 0.451 | (0.283) |
| sexual minority ego | 0.028 | (0.103) | 0.327^∗∗∗^ | (0.088) | 0.656^∗^ | (0.331) |
| same sexual orientation | 0.075 | (0.069) | 0.117^ǂ^ | (0.062) | 0.021 | (0.134) |
| homophobic attitudes alter | –0.008 | (0.018) | 0.036^∗^ | (0.016) | 0.000 | (0.038) |
| homophobic attitudes ego | 0.017 | (0.011) | –0.025^∗^ | (0.013) | –0.024 | (0.033) |
| homophobic attitudes similarity | 0.069 | (0.127) | –0.045 | (0.124) | –0.356 | (0.303) |
| friendship | 0.719^∗∗∗^ | (0.199) | –0.234 | (0.294) | 0.140 | (0.414) |
| reciprocity with friendship | 0.042 | (0.207) | 0.366 | (0.251) | –0.094 | (0.381) |
| outdegree (sqrt) friendship activity | –0.126^∗∗∗^ | (0.029) | –0.124^∗∗∗^ | (0.032) | –0.287^∗∗^ | (0.090) |
| friendship to agreement | 0.661^∗∗∗^ | (0.115) | 0.764^∗∗∗^ | (0.051) | 0.301^∗∗^ | (0.103) |
| *Homophobic attitudes dynamics:* |  |  |  |  |  |  |
| rate homophobic attitudes period 1 | 2.618 | (0.281) | 2.601 | (0.194) | 2.931 | (0.560) |
| linear shape | 0.056 | (0.055) | 0.041 | (0.056) | –0.119 | (0.107) |
| quadratic shape | –0.040 | (0.063) | 0.006 | (0.059) | 0.079 | (0.088) |
| average similarity (friendship) | 4.359^∗^ | (2.118) | 2.348^ǂ^ | (1.308) | 5.543^∗^ | (2.681) |
| average similarity (dislike) | –8.244 | (5.020) | 0.043 | (3.475) | –0.741 | (4.685) |
| effect from gender | –0.292 | (0.183) | –0.221^ǂ^ | (0.129) | –0.155 | (0.244) |
| effect from ethnicity | –0.110 | (0.116) | 0.079 | (0.117) | –1.677 | (1.194) |
| effect from sexual minority orientation | –0.647^ǂ^ | (0.332) | –0.454^∗^ | (0.223) | -0.500 | (fixed)^a^ |

Notes. ^ǂ^ p<0.1; * p<0.05; ** p<0.01, two-sided.

All convergence t-ratios<|0.08|, overall maximum convergence ratio models all smaller than 0.21.

^a^ : *effect from sexual minority orientation* fixed at -0.5 in School 3 (value similar to that observed in Schools 1 and 2) to aid model convergence

# Appendix E. Selected Results Respecified Friendship Selection

In response to the inconsistent and somewhat surprising results with regard to how similarity in homophobic attitudes was related to friendship dynamics, we reran our analysis, this time using a more elaborate specification of how homophobic attitudes might have affected friendship dynamics, based on recent recommendations by Snijders and Lomi (2019) regarding the specification of selection functions in positive social networks. They argue that for positive social networks and scale level attributes, such as friendship networks and homophobic attitudes, homophily tendencies might be confounded with three other, related mechanisms: attachment conformity, which in this case translates to a higher likelihood of becoming or staying friends with a peer, if this peer expresses a normative level of homophobic attitudes; aspiration, here meaning that more homophobic attitudes increase the likelihood of receiving friendship nominations; and sociability, here referring to individuals with more homophobic attitudes being more likely to establish or maintain friendships.

Whenever one suspects that, in addition to homophily, one of these three additional mechanisms might also affect friendship selection, Snijders and Lomi (2019) suggest to test a four-variable selection specification that allows for an identification of all four of these mechanisms. For our data, attachment conformity could be a relevant confounder. Adolescent research has often typified the peer context as an environment that enforces norm-conforming and socially punishes non-conforming behavior (Wright et al. 1986). In line with this, norm-conforming behavioral tendencies have been detected for a wide range of outcomes, including not only antisocial behaviors such as risk taking or bullying (Dijkstra et al. 2008; Santor et al. 2000), but also academic behavior (Bursztyn et al. 2015). In case homophobic (or homosexuality-accepting) attitudes would be the norm, friendship selection on homophobic attitudes could additionally be driven by aspiration mechanisms.

In view of the above, we re-estimated the models, this time using the four variable selection function (Snijders and Lomi 2019) for specifying friendship selection on homophobic attitudes. This specification consists of an *alter*, *squared alter*, *ego*, and and *(ego minus alter) squared* effect, potentially extended with a *squared ego* effect. We tested the four variable specification. A selection of the results is provided in Table E1 and Figures E1-E3, which plot the friendship selection function with varying levels of homophobic attitudes of adolescents (homophobic attitudes ego) and those of their peers (homophobic attitudes alter).

For a detailed explanation and example on how to interpret the implemented four (or five) variable model specification, we refer the interested reader to Snijders and Lomi (2019). Overall, as was the case in our original model specification, also when using the four variable model specification, the extent to and way in which homophobic attitudes affected friendship selection varied considerably from school to school. For evaluating whether homophobic attitudes affected friendship selection at all, we conducted a joint χ^2^ test on all four selection parameters, testing the null hypothesis that all four effect were zero (Ripley et al. 2019). The tests returned a non-significant result in School 1 ($\chi_{4}^{2}$ = 2.91, p=.57), but not in Schools 2 ($\chi_{4}^{2}$ = 14.15, p<.01) and 3 ($\chi_{4}^{2}$ = 11.67, p<.05).

Results also varied when looking at homophily tendencies, which are represented by the *(ego minus alter) squared* effect in this model specification, with negative effects being indicative of homophily (Snijders and Lomi 2019). This effect was close to 0 and non-significant in school 1, indicative of homophily in school 2 (*par(SE)* = –0.012(0.004), p<.01), and indicative of a negative homophily tendency in school 3 (referring to friendship selection or maintenance being less likely with similar levels of homophobic attitudes), (*par(SE)* = 0.025(0.008), p<.01).

Lastly, we shortly evaluate Figures E1 to E3. The Figures confirm the picture of inconsistent results. In School 1 (Figure E1), homophobic attitudes hardly seem to affect friendship selection, the selection function being fairly close to zero for all respondents, varying with neither adolescents’ own levels of homophobic attitudes, nor those of their peers. In Schools 2 and 3, friendship selection related to homophobic attitudes seems to be predominantly affected by homophily tendencies, but opposite ones. In School 2 (Figure E2), adolescents were more likely to select peers as friends when they were similar in homophobic attitudes. In School 3, however, opposites seemed to attract: Adolescents with low (high) levels of homophobic attitudes were more likely to select peers as friends when these peers had high (low) levels of homophobic attitudes.

Table E1. Selected results RSiena models with extended selection function friendship network

|  | School 1 |  | School 2 |  | School 3 |  |  |  |
| --- | --- | --- | --- | --- | --- | --- | --- | --- |
| Effect | parameter | (*SE*) | parameter | (*SE*) | parameter | (*SE*) | Fisher <0 | Fisher >0 |
| *Friendship dynamics:* |  |  |  |  |  |  |  |  |
| homophobic attitudes alter | 0.002 | (0.012) | –0.035ǂ | (0.019) | 0.017 | (0.040) | 0.176 | 0.688 |
| homophobic attitudes squared alter | –0.002 | (0.006) | 0.004 | (0.007) | 0.014 | (0.021) | 0.785 | 0.407 |
| Homophobic attitudes ego | 0.018 | (0.011) | –0.016 | (0.016) | 0.021 | (0.032) | 0.622 | 0.172 |
| Homophobic attitudes difference squared | 0.001 | (0.003) | –0.012** | (0.004) | 0.025** | (0.008) | 0.035 | 0.016 |

Note. ̶ ǂ p<0.1; * p<0.05; ** p<0.01, two-sided.

All convergence t-ratios<|0.07|, overall maximum convergence ratio models all smaller than 0.21.


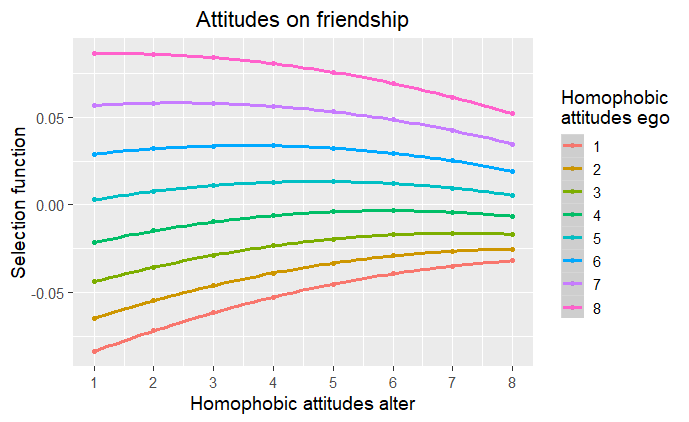


Figure E1. Friendship selection function by homophobic attitudes adolescents (‘Ego’) and their peers (‘Alter’), School 1
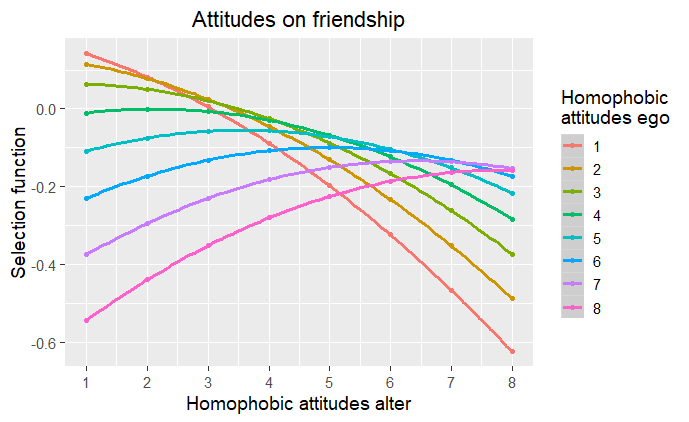
Figure E2. Friendship selection function by homophobic attitudes adolescents (‘Ego’) and their peers (‘Alter’), School 2


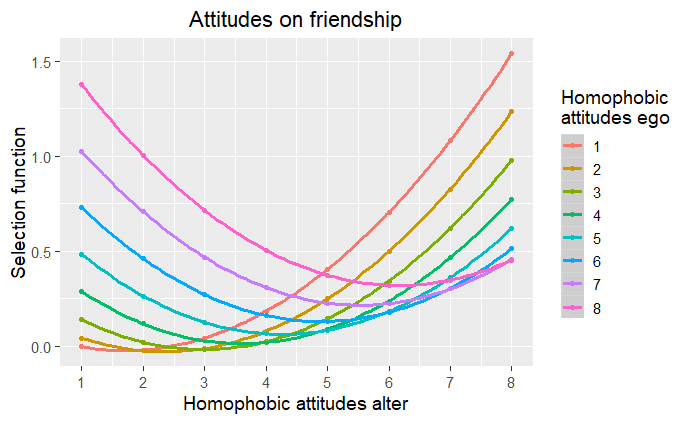


**Figure E3. Friendship selection function by homophobic attitudes adolescents (‘Ego’) and their peers (‘Alter’), School 3**

# References

Bursztyn, L., Jensen, R., Ashraf, N., Dal Bó, E., Linden, L., Mahajan, A., et al. (2015). How does peer pressure affect educational investments? *Quarterly Journal of Economics*.

Costa, A., Bandeira, D., & Nardi, H. (2013). Systematic review of instruments measuring homophobia and related constructs. *Journal of Applied Social Psychology*, *43*(6), 1324–1332. https://doi.org/10.1111/jasp.12140

Dijkstra, J. K., Lindenberg, S., & Veenstra, R. (2008). Beyond the class norm: Bullying behavior of popular adolescents and its relation to peer acceptance and rejection. *Journal of Abnormal Child Psychology*, *36*(8), 1289–1299. https://doi.org/10.1007/s10802-008-9251-7

Galambos, N., Petersen, A., Richards, M., & Gitelson, I. (1985). The attitudes toward women scale for adolescents (AWSA): A study of reliability and validity. *Sex Roles*, *13*, 343–356.

Heider, F. (1946). Attitudes and cognitive organization. *The Journal of Psychology*, *21*, 107–112.

Herek, G. M. (1988). Heterosexuals’attitudes toward lesbians and gay men: Correlates and gender differences. *The Journal of Sex research*, *25*(4), 451–477. https://doi.org/10.1080/00224498809551476

Huisman, M., & Snijders, T. A. B. (2003). Statistical analysis of longitudinal network data with changing composition. *Sociological Methods & Research*, *32*(2), 253–287. https://doi.org/10.1177/0049124103256096

Huisman, M., & Steglich, C. E. G. (2008). Treatment of non-response in longitudinal network studies. *Social Networks*, *30*(4), 297–308. https://doi.org/10.1016/j.socnet.2008.04.004

Krause, R. W., Huisman, M., & Snijders, T. A. B. (2018). Multiple imputation for longitudinal network data. *Italian Journal of Applied Statistics*, *30*(1), 33–57. https://doi.org/10.26398/IJAS.0030-002

Kuyper, L. (2015). *Jongeren en seksuele oriëntatie. Ervaringen van en opvattingen over homoseksuele, biseksuele en heteroseksuele jongeren [Youth and sexual orientation. Experiences of and attitudes towards homosexual, bisexual, and heterosexual youth]*. Den Haag, the Netherlands: Netherlands Institute for Social Research/SCP.

Poteat, V. P., & Espelage, D. (2005). Exploring the relation between bullying and homophobic verbal content: The Homophobic Content Agent Target (HCAT) Scale. *Violence and victims*, *20*, 513–528.

Ripley, R. M., Snijders, T. A. B., Boda, Z., Vörös, A., & Preciado, P. (2019). *Manual for RSiena version 4.0 (version April 9, 2019)*. Oxford, UK: University of Oxford.

Santor, D. A., Messervey, D., & Kusumakar, V. (2000). Measuring peer pressure, popularity, and conformity in adolescent boys and girls: Predicting school performance, sexual attitudes, and substance abuse. *Journal of Youth and Adolescence*, *29*(2), 163–182. https://doi.org/10.1023/A:1005152515264

Snijders, T. A. B., & Lomi, A. (2019). Beyond homophily: Incorporating actor variables in statistical network models. *Network Science*, *7*(1), 1–19. https://doi.org/10.1017/nws.2018.30

Snijders, T. A. B., Lomi, A., & Torló, V. J. (2013). A model for the multiplex dynamics of two-mode and one-mode networks, with an application to employment preference, friendship, and advice. *Social Networks*, *35*, 265–276. https://doi.org/10.1016/j.socnet.2012.05.005

Steglich, C. E. G., Snijders, T. A. B., & Pearson, M. A. (2010). Dynamic networks and behavior: Separating selection from influence. *Sociological Methodology*, *40*(1), 329–393. https://doi.org/10.1111/j.1467-9531.2010.01225.x

van de Meerendonk, B., Eisinga, R., & Felling, A. (2003). Application of Herek’s attitudes toward lesbians and gay men scale in the Netherlands. *Psychological Reports*, *93*(1), 265–275. https://doi.org/10.2466/pr0.2003.93.1.265

van Wijk, E., van De Meerendonk, B., Bakker, F., & Vanwesenbeeck, I. (2005). Moderne homonegativiteit: De constructie van een meetinstrument voor het meten van hedendaagse reacties op zichtbare homoseksualiteit in Nederland [Modern homonegativity: The construction of a measurement instrument for measuring current day reactions to visible homosexuality in the Netherlands]. *Tijdschrift voor seksuologie*, *29*, 19–27.

van Zalk, M. H. W., Kerr, M., van Zalk, N., & Stattin, H. (2013). Xenophobia and tolerance toward immigrants in adolescence: Cross-influence processes within friendships. *Journal of Abnormal Child Psychology*, *41*(4), 627–639. https://doi.org/10.1007/s10802-012-9694-8

Velasco González, K., Verkuyten, M., Weesie, J., & Poppe, E. (2008). Prejudice towards Muslims in The Netherlands: Testing integrated threat theory. *British Journal of Social Psychology*, *47*(4), 667–685. https://doi.org/10.1348/014466608X284443

Wright, J. C., Giammarino, M., & Parad, H. W. (1986). Social status in small groups: Individual-group similarity and the social “misfit”. *Journal of Personality and Social Psychology*, *50*(3), 523–536. https://doi.org/10.1037/0022-3514.50.3.523

1. In addition to the results depicted here, we estimated an exploratory factor analysis using all available items (leading to large dropout) and a confirmatory factor analysis on all items using full information maximum likelihood. Results in terms of factor loadings were similar to the ones presented here. [↑](#footnote-ref-1)
2. https://www.cbs.nl/nl-nl/achtergrond/2016/47/bevolking-naar-migratieachtergrond [↑](#footnote-ref-2)
